# Text mining of practical disaster reports: Case study on Cascadia earthquake preparedness

**DOI:** 10.1371/journal.pone.0313259

**Published:** 2025-01-07

**Authors:** Julia C. Lensing, John Y. Choe, Branden B. Johnson, Jingwen Wang

**Affiliations:** 1 Department of Industrial & Systems Engineering, University of Washington, Seattle, WA, United States of America; 2 Decision Science Research Institute, Inc., Springfield, OR, United States of America; 3 Decision Research Center, Oregon Research Institute, Springfield, OR, United States of America; 4 Department of Computer Science, Elizabethtown College, Elizabethtown, PA, United States of America; The University of Lahore, PAKISTAN

## Abstract

Many practical disaster reports are published daily worldwide in various forms, including after-action reports, response plans, impact assessments, and resiliency plans. These reports serve as vital resources, allowing future generations to learn from past events and better mitigate and prepare for future disasters. However, this extensive practical literature often has limited impact on research and practice due to challenges in synthesizing and analyzing the reports. In this study, we 1) present a corpus of practical reports for text mining and 2) introduce an approach to extract insights from the corpus using select text mining tools. We validate the approach through a case study examining practical reports on the preparedness of the U.S. Pacific Northwest for a magnitude 9 Cascadia Subduction Zone earthquake, which has the potential to disrupt lifeline infrastructures for months. To explore opportunities and challenges associated with text mining of practical disaster reports, we conducted a brief survey of potential user groups. The case study illustrates the types of insights that our approach can extract from a corpus. Notably, it reveals potential differences in priorities between Washington and Oregon state-level emergency management, uncovers latent sentiments expressed within the reports, and identifies inconsistent vocabulary across the field. Survey results highlight that while simple tools may yield insights that are primarily interpretable by experienced professionals, more advanced tools utilizing large language models, such as Generative Pre-trained Transformer (GPT), offer more accessible insights, albeit with known risk associated with current artificial intelligence technologies. To ensure reproducibility, all supporting data and code are made publicly available (DOI: 10.17603/ds2-9s7w-9694).

## Introduction

The Cascadia Subduction Zone (CSZ) that stretches 700 miles from Vancouver Island, Canada to Northern California is capable of producing up to a 9.0 magnitude (M9) megathrust earthquake [1] that would cause expansive damage and disruption across southern British Columbia, Washington, Oregon and north California [2]. As the interval since the last M9 earthquake in 1700 exceeds the average interval between CSZ events, local, state, regional, and federal emergency management professionals have conducted planning conferences and emergency response exercises to increase awareness of potential impacts and build preparedness for an M9 Cascadia megathrust earthquake. Emergency management practitioners capture critical lessons learned, actions taken, and planning considerations from these practical events in after-action reports, response plans, impact assessments, resiliency plans, and other practical reports. These reports are then shared across the field to inform future preparedness events and expand the knowledge base.

These documents are often lengthy and packed with dense information across a wide range of topics and fields which can make them difficult for a reader to digest. They also are usually geared towards a specific disaster event, locality, or infrastructure, which can discourage a reader from reviewing them when it does not directly relate to their area of interest. Though these reports contain a wealth of information, individually they likely have limited readership due to such issues. We propose examining these practical reports as a corpus (body of work as a whole) in order to increase their accessibility and applicability to a wider audience. Examining a corpus may uncover common trends and themes amongst the vast literature, distinctive features that set one document apart from another, emerging patterns throughout a period of time, relationships and connections between documents or represented entities, or underlying emotions across the corpus. Examining a corpus as an aggregate of information-rich individual reports may reveal insights not visible from examining single documents.

To do this, the reader must be able to efficiently cull through hundreds of pages of text and systematically extract and understand important information. Reading and synthesizing even one of these reports is a lengthy and cumbersome process; conducting manual contextual analysis of several reports at once can be out of reach for busy researchers and practitioners. Text mining using statistical software can effectively and efficiently extract statistical and linguistic features from a collection of documents to look for the presence or absence of patterns, trends, associations, distributions, and sentiments [3]. In this paper we present a suite of text mining tools that represents the breadth of existing capabilities, and the tools can be easily implemented with minimal coding experience.

We provide a flexible script of all the presented tools that can be tailored and applied to a variety of document collections. Researchers who may grasp domain-related theory but have little or no coding experience could use such tools to define and understand the research-practice gap by examining what does and does not appear in such application-oriented corpora. Practitioners may also benefit from the suite of tools to gain objective insights into a corpus, and thus complement their traditional research and other information gathering approaches (e.g., literature review, environmental scan, landscape analysis). Our suite of tools can be customized to the practitioner’s preferences, serving as a valuable time-saving tool to gain high-level productive comprehension of the large amounts of data in a variety of corpora.

The main contributions of this work are 1) the presentation of a novel practical report corpus that has not previously been explored using text mining, 2) an approach to extract insights from the corpus using select text mining tools, and 3) a flexible script that researchers and practitioners can apply to a variety of corpora. We validate our approach by implementing our suite of tools on our case study corpus, and conduct a brief survey of emergency management professionals to elicit feedback on our proposed approach. Lastly, we identify key insights from the individual tools presented and then synthesize our findings to present practical implications for emergency management and other risk professionals, including researcher and practitioner user groups.

The remainder of the paper is organized as follows: Background provides an overview of text mining procedures and applications. Approach details the procedures for developing our corpus, and describes the text mining tools’ use in our approach. In Case study: Application, results, and interpretations, we present a case study of the application of the approach along with results and their interpretations, and findings from our informal survey. Finally, we conclude in Discussion with strengths and limitations of our proposed approach, implications for our identified user groups, and recommendations for future work.

## Background

### Foundations and modern advances in text mining

In broad terms, text mining is a “knowledge-intensive process in which a user interacts with a document collection over time using a suite of analysis tools” [3]. The available tools, techniques, algorithms, and applications of text mining continue to grow in size and scope as the development and availability of web-based textual information continues to grow [4]. Text mining involves techniques from natural language processing (NLP), machine learning, statistical analysis, and artificial intelligence (AI) to extract meaningful information from dense, unstructured textual data. It is used across a wide variety of domains and applications including information retrieval, content analysis, social media monitoring, market research, customer feedback analysis, fraud detection, and healthcare analysis. Regardless of the applied tool, text mining involves the same steps. It begins with identifying the document collection. These corpora vary widely in size, content, and format, and may cover a broad range of topics or be focused on a specific domain or subject. The ever-growing textual information found on web-based media has been supplemented by development of other collections, such as websites, emails, and social media posts [4]. The next step is to convert textual, unstructured data from the corpus into structured, machine-readable elements. This involves preprocessing operations such as removing irrelevant information for the task at hand (removing numbers or ‘stop words’), grouping together different inflected forms of the same word (lemmatization), or correcting any grammatical or contextual errors (misspellings, abbreviations, case correcting, etc.) [5]. Next, the remaining textual information for each document must be formulated into a numerical representation of each document. The most commonly used representations are Bag-of-Words (BoW), tf-idf (combination of the word’s frequency in a document with its rarity across all documents) [6] and, more recently, *word embedding* [7]. Once textual data are preprocessed and converted into a suitable format, various analytical tools can be applied. These include categorization and classification (assigning documents to predefined categories or labels) [8], clustering (grouping similar documents together) [9], topic modeling (identifying latent topics within the text), sentiment analysis (understanding the sentiment or opinion expressed in the text), summarization (creating a short, coherent representation of a larger document) [10], among many others. Finally, the results are evaluated for quality and relevance and then synthesized with background knowledge/expertise to develop actionable insights.

In this work we follow these established procedures, adhering to accepted practices in the field to guide our analysis. However, as the tools for text mining evolve, particularly with the integration of advanced artificial intelligence (AI) models, the ability to extract deeper, more comprehensive insights from unstructured textual data has become increasingly more robust. Recent advancements in Large Language Models (LLMs), Deep Learning, and Natural Language Processing(NLP) further enhanced the traditional text mining process, offering new approaches to real-time data analysis and text interpretation, which are particularly relevant in disaster management contexts. For example, the development of LLM-assisted platforms enables faster, more efficient crisis management, allowing for real-time collaboration and decision-making during emergencies [11]. In parallel, deep learning methods for tweet classification have been applied to prioritize and schedule rescue efforts by classifying critical information from social media posts during Hurricanes Harvey and Irma [12]. Additionally, hybrid and ensemble models in NLP have further improved the accuracy of common text mining tasks such as classification, clustering, and sentiment analysis, making these tools increasingly valuable for analyzing disaster reports [13].

While these advancements demonstrate the potential of modern AI techniques, text mining remains a foundational approach that effectively complements these new tools. By utilizing established text mining tools, we can systematically extract valuable insights from large volumes of unstructured data, providing a robust framework for analysis. Text mining is particularly well-suited for exploring specific nuances and contextual details found in disaster practical reports, which may be overlooked by more generalized AI models. Therefore, rather than making traditional text mining obsolete, these advancements enhance its effectiveness, enabling a more in-depth and detailed understanding of the text. The integration of established methods with innovative technologies can equip researchers and practitioners with the tools needed to address the complexities inherent in disaster management.

### Motivation for exploring disaster preparedness practical reports

In this study, we present a corpus of practical reports related to emergency preparedness for a Cascadia M9 megathrust earthquake. It is important to analyze this corpus because it represents the unique perspective of emergency management practitioners and can aid in bridging the knowledge gap between research and practice. [14] identify “a need for synthesis and compilation of lessons learned.” Doing so can lead to innovation and change across the emergency management community. Specifically, for public health emergency preparedness and response (PHEPR), [15] call for “a national repository of [after-action reports (AARs)] or of reports based on analysis of AARs” for dissemination of lessons learned to support PHEPR practices and demonstrate a labor-intensive method to rigorously synthesize research studies and reports (e.g., AARs, case reports). Additionally, a body of knowledge from the practitioner’s perspective represents factors that can be difficult to be represented in research (e.g., experience, relationships, etc.) and have been found to be significant determinants of success during disaster recovery [16]. Finally, [17] make distinctions between the strategic, operational, and tactical levels of an emergency response system and identifies their unique requirements and contributions to effective disaster recovery practices. Both perspectives are valuable but differ in size, scope, and impact. By extracting valuable insights from the practitioner, we contribute to the marrying of these two perspectives to ensure continuity and unity at all echelons [18].

This study presents an effective and efficient method to compile important insights, knowledge, and lessons learned from the practitioners’ viewpoint.

### Relevant corpora that have been explored using text mining

In the emergency management field, text mining is increasingly used to glean information from scientific and academic corpora. Text mining has been used to identify important aspects of resilience in emergency management [19], determine critical success factors in disaster management [20], and analyze resiliency of critical infrastructures [21, 22].

The introduction of social media platforms such as Facebook, Twitter, and Instagram precipitated the development of a new category for corpora. Text mining has been used in disaster recovery by extracting locations and failure intensities of infrastructure during the 2015 flood in Chennai, India [23], examining infrastructure-related status [24] and underlying public emotion post-disaster [25] from Twitter data, surveying public sentiment from Tweets following 2018 earthquakes in Lombok and Bali [26], and examining social cohesion of impacted populations following major hurricanes Harvey, Irma, and Maria [27].

More recently, text mining tools have been applied to other collections of government reports to quickly synthesize general concepts from large textual data sets. [28] applied topic modeling and content analysis of a corpus of urban resilience plans to efficiently extract important information pertaining to climate change and urban planning. [29] explored a corpus of COVID-19 reports using feature extraction to understand the global environment surrounding COVID-19 management, and [30] used text mining to understand emotions surrounding the suspension of a COVID-19 vaccine. Other researchers applied text mining tools to a corpus of annual business reports (10-K) to assess organizational cybersecurity attitudes [31].

Perhaps the most similar to our proposed corpus is that of accident reports. Researchers in the risk analysis field have employed text mining tools to evaluate common cause factors for coal mining accidents [32], pipeline incidents [33, 34], and rail accidents [35].

In our study, we explore a new corpus of practical reports and demonstrate the ability to extract insights from the unique perspective of the practitioner.

## Approach

In this section, we outline the scope of our corpus of practical reports and then discuss the details of our document and feature analysis, sentiment analysis, and topic modeling and summarization. We conclude with our survey protocols.

### Corpus development

As discussed above, many different corpora have been used for text mining, but there has been limited exploratory work of practical reports as a corpus. Practical reports are used across various fields (e.g. health sciences, military, engineering, emergency/disaster management) to document procedures, findings, and outcomes of specific experiments, practical activities, or other hands-on exercises. In the emergency management field, common practical reports include after-action reports, resiliency assessments, response plans, and planning scenarios. While each of these reports serves a distinct function, they contain interconnected elements of a comprehensive approach to enhancing emergency preparedness, response, and overall resilience. We use this set of documents and the definitions below to establish the scope of our corpus.

After-action reports (AARs) evaluate a completed activity, mission, or event and capture lessons learned, identify strengths and weaknesses, and make recommendations for future improvement. Resiliency assessments evaluate an organization’s capacity to withstand and recover from adverse situations, challenges, or crises, and measure various factors that contribute to resilience. A response plan documents procedures, actions, and protocols designed to guide individuals, organizations, or communities in effectively managing and responding to emergencies, crises, disasters, or other unexpected events. A planning scenario is a hypothetical situation or set of circumstances used in the development of plans, strategies, or exercises to prepare for and respond to emergencies, crises, or other events.

### Suite of tools for text mining analysis

Below, we discuss our suite of text mining tools. Our suite of tools can be applied to any practical report corpus to gain overall understanding of its makeup, represent its sentiment, and identify its common themes and ideas. This approach provides a baseline, exploratory overview of a corpus that serves as a jumping-off point for more detailed follow-on analysis, and can help answer our research question: *What are the unique or generalizable characteristics in this corpus that represent the relevant past efforts in practice?*

#### Document and corpus feature analysis

In document and corpus feature analysis, we explore the individual terms that form a document. We can examine terms at the document level or aggregate them at the corpus level. The tools shown here help us answer questions like *what are the predominant themes in this corpus?* and *what are the unique or characteristic themes in each document of the corpus?*. We can also use these tools to compare terms between documents.

To conduct feature analysis, we use a Bag-of-Words (BoW) model that transforms each document into a vector such as *v* = [*x*_1_, *x*_2_, …, *x*_*n*_], where *x*_*i*_ represents a term frequency or term frequency-inverse document frequency (tf-idf) [36]. The term frequency is a count of the occurrence of words in a single document. Commonly, words with high frequency represent the general concepts and ideas within the document. Tf-idf helps the researcher to identify the important words in each document by placing heavier weight on the words that are used less frequently in each document and decreasing the weight for words or terms that are used frequently and commonly throughout the corpus [37]. It is represented by

$$\begin{array}{c} \text{tf}-\text{idf}\left(w,d\right)=\text{TermFreq}\left(w,d\right)\cdot \text{log}(\frac{N}{\text{DocFreq}(w)}), \end{array}$$ (1)

where TermFreq(*w*, *d*) is the frequency of the word *w* in the document *d*, *N* is the total number of documents, and DocFreq(*w*) is the number of documents containing the word *w* [3]. In this analysis, we explore these vector representations as is and in more complex modeling efforts described below.

#### Sentiment analysis

Using our vector representations from feature analysis, we can also explore the sentiment or feeling across the corpus and answer the question *what is the portrayed sentiment of this document or corpus?*

In this work we use two general-use lexicons—Bing and NRC. The Bing Liu and collaborators (Bing) lexicon categorizes English words into a binary category for negative or positive sentiment and conducts a count of those terms that have the greatest contribution to overall sentiment [38]. Similarly, the National Research Council (NRC) lexicon [39, 40] associates words with two sentiments (negative and positive) but also categorizes terms into eight basic emotions (anger, fear, anticipation, trust, surprise, sadness, joy, and disgust) [41].

#### Topic modeling and summarization

Topic modeling assigns a collection of texts into natural groupings to better understand the whole collection, aid in uncovering hidden themes from within a collection, assigning documents to discovered themes, or using these assignments to further organize or summarize the collection [42]. It can aid in answering the questions *what are the most common topics discussed in the corpus?* and *which documents comprise identified topics*. Text summarization involves creating a short, coherent representation of a larger document or corpus by distilling the most important information from a source [10] and can be used to answer the question *how can you summarize this content?*

In this work, we conduct topic modeling using Latent Dirichlet Allocation (LDA) and Bidirectional Encoder Representations from Transformers (BERT). Finally, we utilize Generative Pre-trained Transformers (GPT) models to conduct text summarization on identified topics.

*Latent Dirichlet Allocation (LDA)*. Latent Dirichlet Allocation (LDA) is a generative probabilistic model that is commonly used in topic modeling and describes each document in the corpus as a collection of topics and each topic as a collection of words. LDA is an iterative process that uses probability distributions to work backward to identify which topics would have generated these documents, and which words would have generated those topics. The number of topics *k* is determined by the researcher and the algorithm iterates over each document *d* and each word *w* to determine the product of the probability that the topic *t* is contained in document *d* and the probability that the word *w* is contained in topic *t* to determine the most relevant topic for the current word. This process continues until a steady state is achieved, rendering the assignment of words *w* to *k*-number of topics based on the probability that a word *w* belongs to a topic *t* [43].

*Bidirectional Encoder Representations from Transformers (BERT) and Generative Pre-trained Transformers (GPT)*. Up to this point, we have discussed single-dimension representation of documents for analysis (term frequency, tf-idf, etc.). While these tools are simple to implement and easily accessible, they do not account for the order or the relationship of terms [44]. Recently, state-of-the-art large language models (LLMs) have gained traction for their ability to incorporate semantic analysis using word or sentence embeddings (dense, multidimensional representation of words or sentences in the form of vectors), and their pre-training on large, general-use data sets that can then be applied broadly to a variety of corpora [45]. Current popular large language models such as BERT and GPT use embeddings to represent documents and then pass them through a transformer model that uses attention to track relationships in sequential data (e.g. words or terms in a sentence or sentences in a paragraph) [46].

For this analysis, we explore BERT tools by implementing BERTopic on our corpus in Python. BERTopic converts documents into vector representations (embeddings) using a sentence transformer. It clusters the embeddings using a clustering algorithm and then generates the topic of each cluster based on the cluster-level tf-idf (c-tf-idf) [47]. Much like tf-idf discussed previously, c-tf-idf extracts the most important words in each cluster (c) as opposed to the entire corpus.

Based on the bag-of-words topic representation produced in BERTopic, we generate a short summary of each topic using GPT tools. Unlike BERT, GPT is an autoregressive model that predicts a term based only on the information to the left of the term [48].

### Evaluation of suite of tools

To support our claim that the proposed suite of tools can be useful for researchers and practitioners, we summarize the case study’s findings in a 9-page white paper which takes less than 10 minutes to read (S1 File). We shared it with researchers (including social scientists and engineers) and practitioners (including emergency managers, consultants, engineers, coordinators, and advisors), with members of both groups having little to extensive knowledge about the M9 event. We asked these researchers and practitioners, recruited through the authors’ professional contacts, to provide anonymous comments through Google Form on 1) the specific aspects of the tools that they found particularly useful and 2) challenges or limitations they could identify. We offered $35 gift cards as participant incentives. This survey was exempt from institutional review board (IRB) review.

## Case study: Application, results, and interpretations

In this section, we apply our suite of tools to a developed corpus of practical reports, present results and visualizations, offer possible interpretations of these results, and discuss our survey findings.

We note that the presented results below are specific to this case study’s corpus, and the results’ interpretations are examples of what researchers and practitioners can gain from applying this approach without aiming to be comprehensive.

Due to space limitations, we describe the most significant findings in the section below. Additional insights can be found in S1 Appendix.

### Corpus development

The motivation for this exploratory work sprang from the sharing of a collection of emergency preparedness reports from social science researchers who studied the lifeline infrastructure recovery that will follow the Cascadia Subduction Zone megathrust earthquake [49]. Valuing the merit of this collection, our team sought to better understand its composition, scope, and distinctiveness through text mining.

Our initial research sample of documents consisted of 27 publicly available reports about disaster preparedness for Cascadia events in Washington, Oregon, and California as well as regional and federal-level planning documents. Of the 27 documents, 21 were retained for initial analysis. We removed any documents that did not contain the richness of text required for analysis (e.g., phone directories, report covers, presentations) and any document that did not meet the inclusion and exclusion criteria below.

On inclusion and exclusion criteria, we included general practical reports about disaster preparedness for Cascadia earthquake events as well as specific reports related to post-Cascadia event infrastructure recovery (e.g. water, power, transportation, and telecommunications). We excluded reports related to hospital or healthcare emergency preparedness (unless coupled with another infrastructure, plus academic research articles, news articles, blog posts, and non-technical articles (e.g. information for the public).

To ensure that the initial sample adequately represented publicly available literature, we conducted a Google search of state, regional, and federal preparedness-related websites for any additional reports that would make our corpus more robust. For state-level practical reports, we searched on Washington, Oregon, and California official government sites related to Cascadia events preparedness (e.g. Washington Emergency Management Division, Oregon Department of Emergency Management, and California Governor’s Office of Emergency Services). We also examined any local or state-affiliated research centers that may produce reports (e.g. Pacific Northwest National Laboratory, Oregon State University Extension Earthquake Preparedness, Cascadia Region Earthquake Workgroup). We manually reviewed identified websites and conducted a search for “Cascadia” on each website’s search bar and then reviewed hits that met inclusion criteria. To ensure that we did not miss any important documents, we also conducted a generalized Google search with relevant keywords (“*state* Cascadia subduction zone disaster preparedness practical reports”) to capture state-level documents. A similar process was conducted for regional and federal entities. We reviewed websites for the Federal Emergency Management Administration (FEMA), National Oceanic and Atmospheric Administration (NOAA), Earthquake Engineering Research Institute (EERI), and the United States Geological Survey (USGS), plus a general Google search. Finally, we conducted a Google search with infrastructure-based keywords (“water”, “utility”, “electricity”, “transportation”, “telecommunication”) to identify any preparedness reports from individual infrastructure entities. This search yielded an additional 17 reports for inclusion in the corpus.

Our corpus is representative of the type of practical reports that are publicly available, and intended to be exhaustive concerning the aforementioned inclusion and exclusion criteria at the time of our search from January to March 2022. We searched manually and took relevance, quality, and complexity into account to determine the comprehensiveness of the corpus. We concluded our search when we stopped discovering new information relevant to our objectives. We also took into account maintaining consistency in our search procedures (e.g. depth of search state-to-state and among infrastructures).

**Fig 1:**
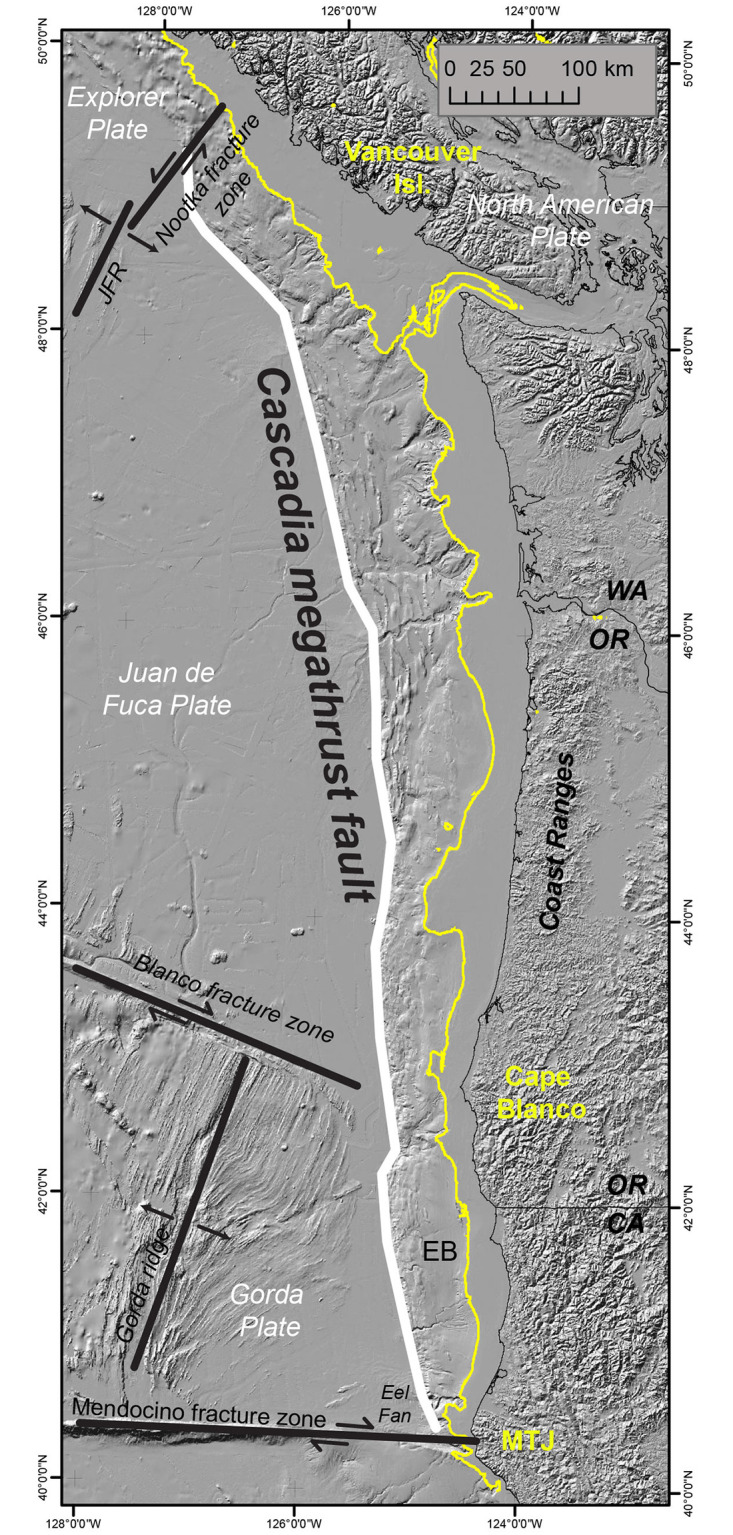
Map of the Cascadia subduction zone [50].

Our final corpus comprises 38 practical reports representing Cascadia earthquake preparedness planning at the state, regional, and federal levels (see Table 1 for summary). There are 29 reports from the state level, 7 from the regional level (encompassing WA, OR, CA), and 2 reports from the federal level. Of the 29 state-level reports, 2 are from California, 21 from Oregon, and 6 from Washington. This numerical difference between the states may be attributed to varying levels of interest, likely because the longest and most central portion of the Cascadia subduction zone abuts Oregon, followed by Washington and California in the U.S. as shown in Fig 1. Given the inclusion criteria and search protocols for our study’s purpose, the over-representation of Oregon is considered fair. The equitable representation of state-level reports can and should be considered for the corpus design process if the study’s purpose is to compare between states. The average page length of the documents in the corpus is approximately 80 pages with the shortest document at 4 pages and the longest document at 341 pages. The oldest document was published in 1997, and the most recent was 2022.

**Table 1:** Corpus summary.

Document	Page Length	Year	Document Type	Level	State
AAR Cascadia Rising WA State	102	2018	After Action Report	State	WA
AAR Cascadia Rising WA State	38	2022	After Action Report	State	WA
AFIT Basing and Resupply Report	69	2017	Response Plan	Federal	-
Analytical Baseline Study for Cascadia Events	324	2011	Impact Assessment	Federal	-
Blue Cascades After Action Report	66	2018	After Action Report	Regional	-
California CSZ and Tsunami Response Plans	144	2013	Response Plan	State	CA
Cascadia Deep Earthquakes	28	2008	Impact Assessment	Regional	-
Cascadia Earthquake Economics Report	17	2020	Impact Assessment	State	OR
Cascadia Subduction Scenario	30	2013	Planning Scenario	Regional	-
CREW Shallow Earthquakes	32	2009	Impact Assessment	Regional	-
CREW Tabletop Exercises	16	2007	Planning Scenario	Regional	-
CSZ Bridge Impact Report	163	2016	Impact Assessment	State	OR
CSZ Hazard Mapping	147	1997	Impact Assessment	State	OR
Earthquake Scientific Consensus	155	2000	Planning Scenario	State	OR
GHPUD Resilience Study Report	37	2020	Resilience Plan	State	WA
Impacts of CSZ Earthquake on CEI Hub	55	2021	Impact Assessment	State	OR
Joint Multi-State AAR Cascadia Rising	46	2016	After Action Report	Regional	-
Oregon Cascadia PlaybookV3	100	2018	Response Plan	State	OR
Oregon Cascadia Rising AAR	60	2016	After Action Report	State	OR
Oregon DOT Highway Report	19	2013	Impact Assessment	State	OR
Oregon Earthquake Report	157	2012	Impact Assessment	State	OR
Oregon Fuel Action Plan	92	2017	Action Plan	State	OR
Oregon Hospital and Water System Earthquake Risk Evaluation	164	2014	Impact Assessment	State	OR
Oregon Infrastructure Report Card	95	2019	Impact Assessment	State	OR
Oregon Resilience Plan Final	341	2013	Resilience Plan	State	OR
Oregon Transportation Systems Resiliency Assessment	135	2021	Impact Assessment	State	OR
OSSPAC CEI-Hub Report	46	2019	Resilience Plan	State	OR
PWRTF After Action Report Summary	4	2021	After Action Report	State	OR
Resilient Infrastructure Planning Exercise	52	2018	Response Plan	State	OR
Resilient Washington State	40	2012	Resilience Plan	State	WA
Seattle Cascadia Earthquake Workshop Interdependencies	19	2017	Impact Assessment	Regional	-
Vigilant Guard After Action Report	69	2017	After Action Report	State	CA
Washington State Fuel Shortage Action Plan	14	2019	Action Plan	State	WA
Washington State Transportation Resiliency Assessment	69	2019	Resilience Plan	State	WA
Water Interconnection Report	18	2010	Action Plan	State	OR
What to Expect—Climate Change and Earthquakes in Portland	8	2017	Impact Assessment	State	OR

### Document and corpus feature analysis

To conduct document and corpus feature analysis, sentiment analysis, and topic modeling using LDA, we use R programming language and ‘tidy’ text principles and tools [37]. We conduct preprocessing operations by accepted practices and standardize the representation of each document in our corpus using vectors. Supporting data and code can be found at [51].

In this section, we explore the individual terms that form a document by examining term frequencies and relationships, and term frequency-inverse document frequency (tf-idf).

#### Term frequencies—What are the predominant themes in this corpus?

**Fig 2:**
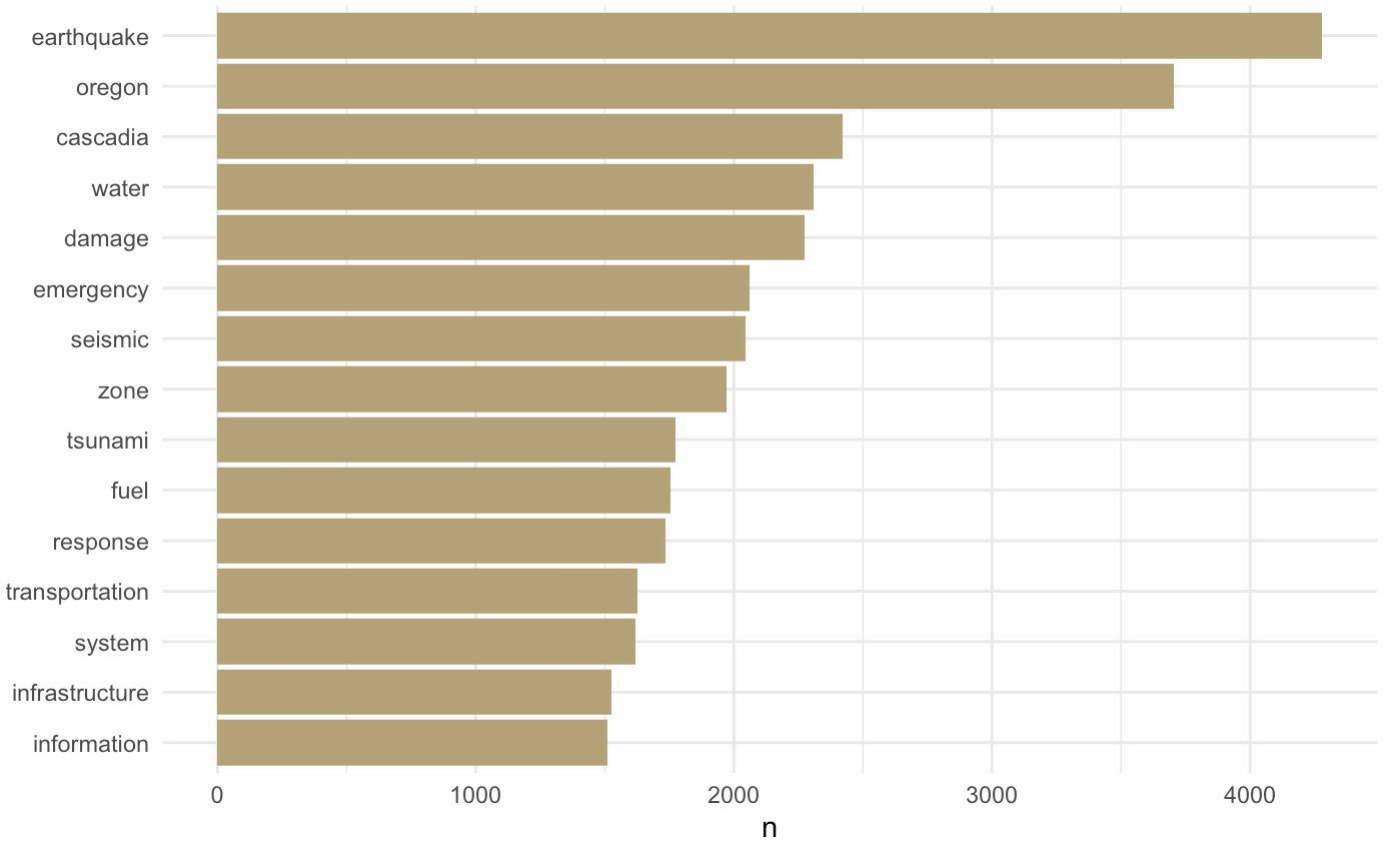
Term frequency—Top 15 most frequently used single-word terms in the corpus.

Fig 2 shows the term frequencies across the corpus, sorted in decreasing order, to summarize predominant themes. Some notable trends offer insights into the corpus. For example, Oregon is the second most frequently mentioned, whereas Washington did not make it to this list of the top 15. This trend is likely because 21 out of 29 state reports are from Oregon versus 6 reports from Washington. Note that a further investigation below the top 15 may be informative, but one should place lesser significance between lower ranks due to Zipf’s law (i.e., a term’s frequency is approximately inversely proportional to the term’s rank).

Water and transportation are the only two infrastructure systems that made it to the top 15. This is surprising because of the commonly perceived importance of the electricity system, upon which other systems’ functionality depends. Telecommunications is notably absent as well and is commonly perceived as a critical infrastructure to facilitate coordination during the immediate disaster recovery period. The higher ranks of *water* and *fuel* over the other infrastructures indicate a greater emphasis on *emergency response* (i.e., additional two terms appearing in the top 15) over recovery. Therefore, the predominant themes in the corpus seem focused on short-term over long-term actions in the aftermath.

These apparent ‘gaps’ in the corpus may warrant further investigation to determine their significance and causes. For instance, do these gaps reflect blind spots in practical implementation, where certain disaster preparedness strategies or information are overlooked by practitioners? Alternatively, do they represent barriers to public access to information, where critical information might not be widely available or easily accessible to relevant stakeholders? Furthermore, these gaps could signal the need to refine the approach by expanding the corpus itself. This could involve using a broader set of keywords, incorporating more diverse data sources, or tapping into less conventional repositories of information to ensure a more comprehensive coverage of disaster preparedness exercises, practices, and insights. Additional exploration may reveal other underlying factors contributing to these gaps, such as the possibility that report writers assume the infrastructure is already sufficiently capable of withstanding significant events, or that certain aspects of infrastructure assessment fall outside their jurisdiction due to a lack of assigned responsibility. The absence of critical infrastructure, like ferry services in Oregon, serves as a narrow example of the latter, highlighting how infrequent considerations may lead to gaps in preparedness discussions.

#### Term relationships—What are the predominant themes in this corpus?

**Fig 3:**
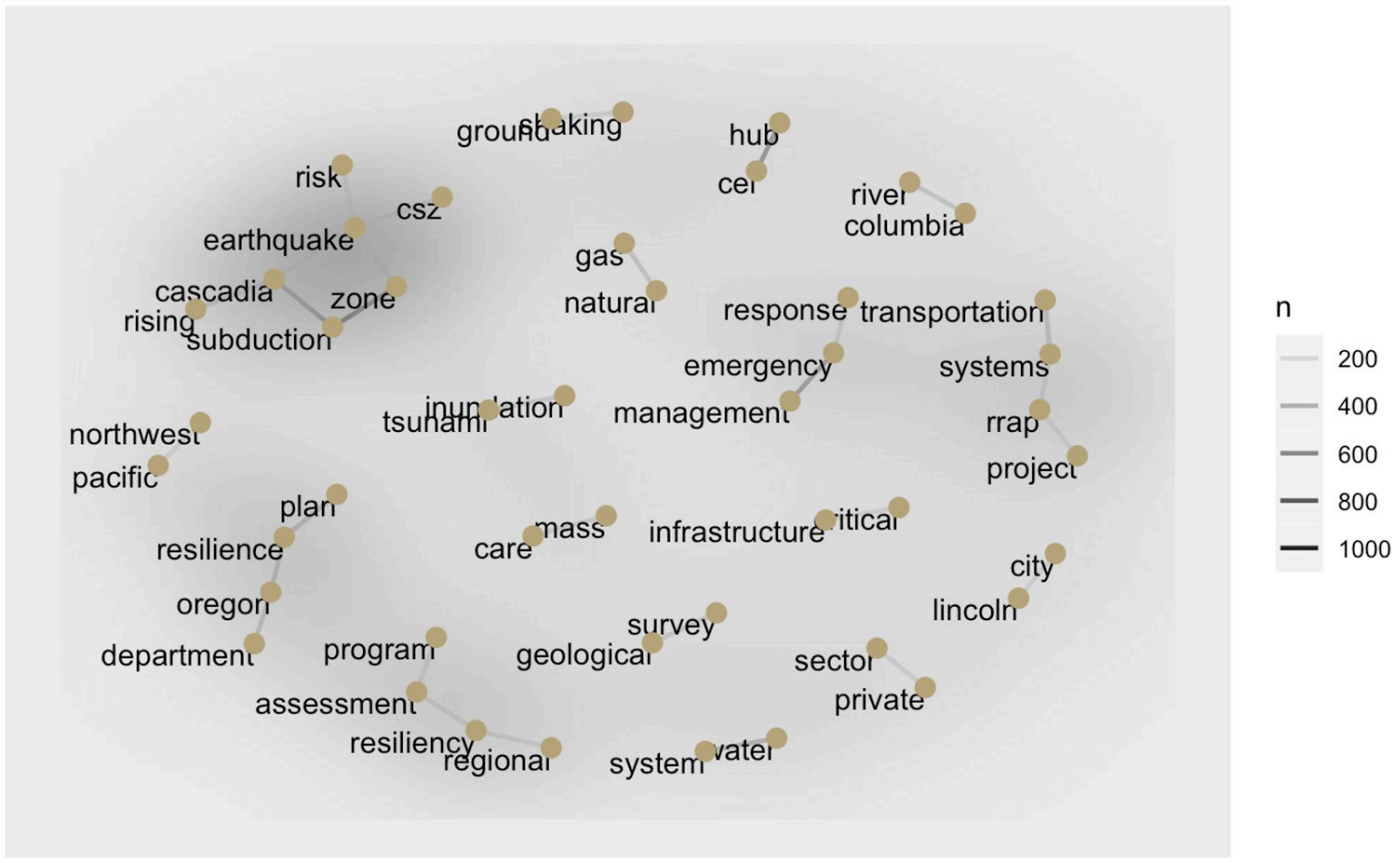
Term relationships—bi-grams (2 sequential and adjacent terms) that occur more than 200 times throughout the corpus and their occurrence with other bi-grams/terms. The opacity of the edge and background represent the frequency of the pair (darker = more frequently used, lighter = less frequently used).

Fig 3 reveals more in-depth insights than Fig 2 into how practical reports use each term in its immediate context. For example, the most frequent terms in Fig 2, such as earthquake, Oregon, and Cascadia, commonly appear in specific contexts, e.g., to denote the hazard at issue, namely, Cascadia subduction zone earthquake, or to discuss Oregon-specific preparedness (e.g., Oregon resilience plan, hospital, fuel, transportation systems). By contrast, Fig 3 highlights the importance of the following bi-grams in M9 preparedness: private sector, natural gas, mass care, Critical Energy Infrastructure (CEI) Hub, Lincoln City, and Columbia River. For example, the corpus shows practitioners’ frequent consideration of the private sector that owns more than 80% of the U.S. critical infrastructure [52]. In sum, these insights could help guide more effective disaster preparedness planning, both at local and national levels, by identifying critical points of intervention and collaboration.

#### Term frequency—Inverse document frequency of whole corpus—What are the unique/characteristic themes in each document of the corpus?

**Fig 4:**
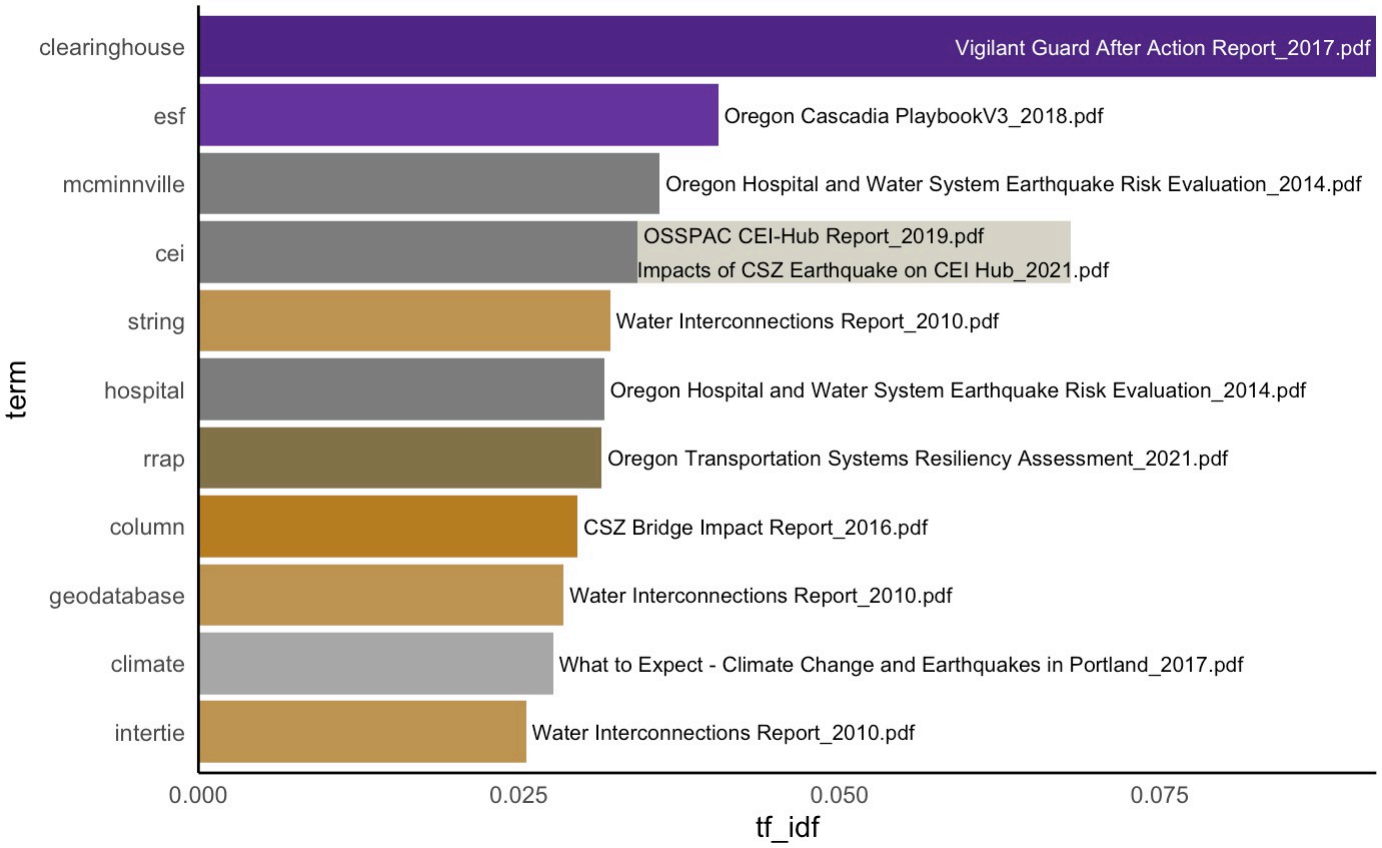
Term frequency—Inverse document frequency of whole corpus. Notable acronyms are as follows. ESF: Emergency Support Function. CEI: Critical Energy Infrastructure. RRAP: Regional Resiliency Assessment Program.

Term frequency and inverse document frequency (tf-idf) represents novel concepts that are particularly emphasized in each practical report. For example, local emergency managers, infrastructure managers, and business continuity practitioners may be surprised to see Fig 4, which reveals that only one or two documents pay attention to some terms widely considered critical in practice (e.g., clearinghouse, ESF [Emergency Support Function], hospital, geodatabase, climate, hub, oil, consortium, substation).

Another noticeable insight is the use of different terms across various documents, such as clearinghouse, hub, and consortium, all of which emphasize the need for coordination to streamline information sharing and response actions. This analysis echoes the well-documented need for standardizing the vocabulary and means for communication in emergency management because the lack thereof is widely recognized as a barrier in information sharing [53]. The identified need for an information clearinghouse is one of the immediate takeaways from this analysis of Cascadia practical reports, but its implications extend beyond this specific context. On a broader scale, the establishment of centralized platforms for information exchange, supported by standardized terminology, is critical for effective disaster response and recovery across various hazard scenarios.

#### Term frequency—Inverse document frequency of select pairs—How do characteristic terms in one document compare to those of another related document?

**Fig 5:**
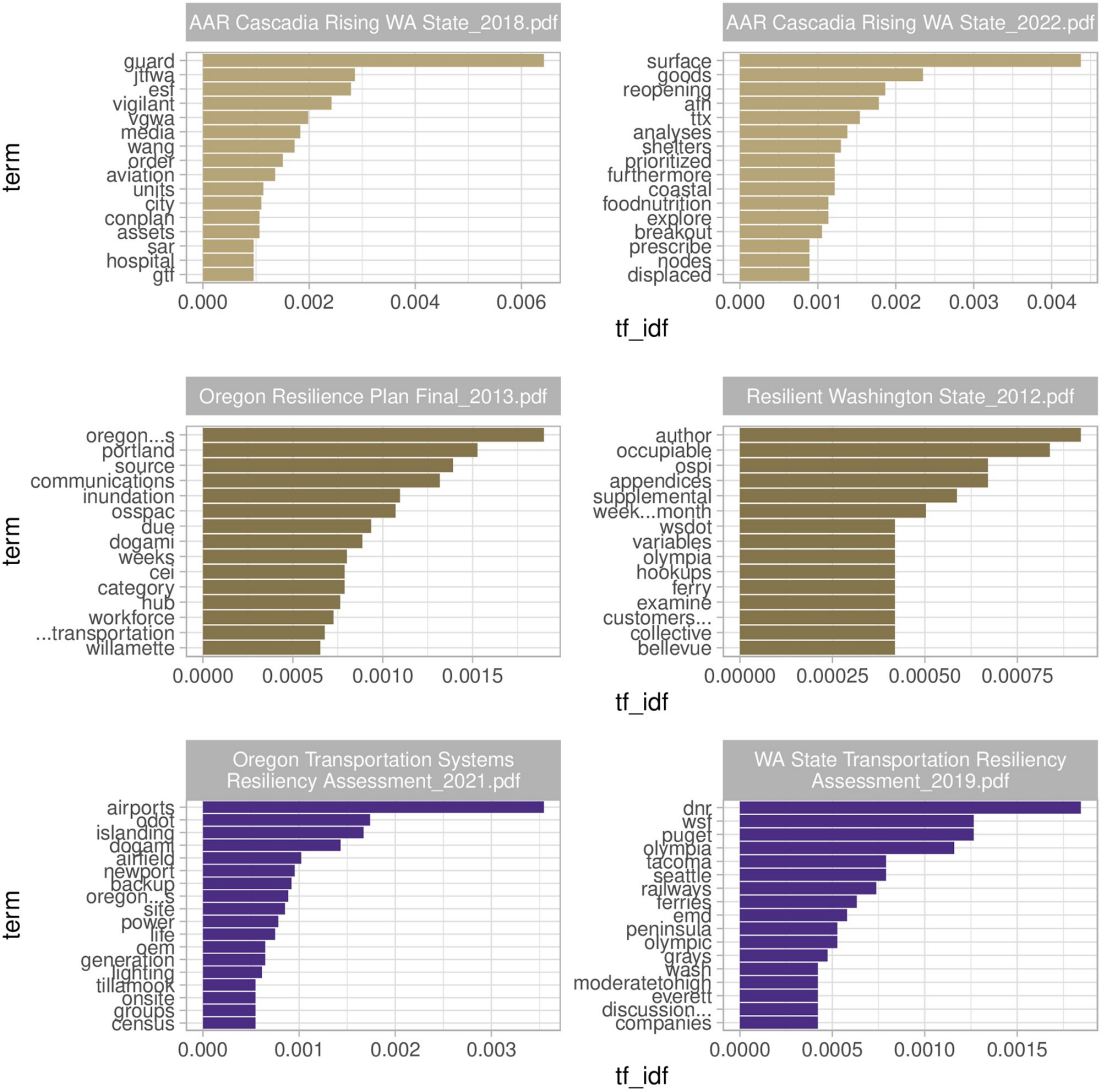
Term frequency—Inverse document frequency of select pairs. Notable acronyms are as follows. JTF-WA: Joint Task Force—Washington. VG-WA: Vigilant Guard—Washington. ESF: Emergency Support Function. WANG: Washington National Guard. CONPLAN: Contingency Plan. SAR: Search and Rescue. GTF: Geographic Task Forces. AFN: Access and Functional Needs. TTX: Table-Top Exercise. OSSPAC: Oregon Seismic Safety Policy Advisory Commission. DOGAMI: Oregon Department of Geology and Mineral Industries. CEI Hub: Critical Energy Infrastructure Hub. OSPI: Office of Superintendent of Public Instruction. WSDOT: Washington State Department of Transportation. ODOT: Oregon Department of Transportation. OEM: Office of Emergency Management. DNR: Department of Natural Resources. WSF: Washington State Ferries. EMD: Emergency Management Division. Note: the length differential between Oregon Resilience Plan (341 pages) and Resilient Washington State (40 pages) explains the plateau of tf-idf for WA; a longer document *d* in Eq 1 tends to have a greater variety of word *w* which does not appear in the rest of the documents considered in TF-IDF. These unique terms with the same frequency create a plateau.

Fig 5 shows characteristic terms in three select pairs of documents. Each term’s “characteristic-ness” within a document is measured by comparing two documents (not the entire corpus), which were identified as similar in their document-topic probabilities (Fig 9): 1) Washington state CSZ event exercise AAR in 2018 vs. 2022; 2) state-level resilience planning in OR vs. WA; and 3) state-level transportation systems study through the Regional Resiliency Assessment Program (RRAP) in OR vs. WA.

The first row in Fig 5 compares WA AARs on Cascadia Rising (CR) in 2016 (the corresponding report was published in 2017 and revised in 2018) and in 2022. The shift of characteristic terms (in *italic* in this and the next few paragraphs) from CR16 to CR22 is noticeable. CR16, which unlike CR22 was integrated with the Washington National Guard (*WANG*)’s *Vigilant Guard* exercise, is characterized by the term *guard* and *SAR* (Search and Rescue). This is consistent with operational findings from this report as SAR after the CSZ earthquake will originate not from typically recognized first responders (e.g., national *guard* members), but by ordinary people in the immediate area and community. CR22 emphasizes the *reopening* of critical transportation (e.g., including *surface* transportation) to deliver lifesaving and life-sustaining bulk *goods* and resource support for “impacted, isolated and supporting communities,” including *coastal* communities. The COVID-19 pandemic appears to have influenced some of the characteristic terms in CR22 (e.g., *shelter*, *reopening*, *AFN*), which demonstrate the collective knowledge and experience gained through the coordinated response to the unprecedented challenges brought by the pandemic since 2020 (i.e., after CR16).

The second row in Fig 5 compares state-level resilience plans: Oregon Resilience Plan (ORP) vs. Resilient Washington State (RWS). tf-idf statistics correctly identify state-specific keywords, such as major geographical names (e.g., Portland and Willamette [valley/river] in OR vs. Olympia and Bellevue in WA) and state agencies in charge of the state resilience (e.g., OSSPAC and DOGAMI in OR vs. OSPI and WSDOT in WA). More interesting findings are ORP’s emphases on *communications, inundation, workforce*, and *transportation* in contrast to RWS’ keywords, such as *occupiable, ferry* and *OSPI*, which is in charge of schools’ resilience.

The third row in Fig 5 compares state-level transportation resiliency. Like the second row, tf-idf statistics correctly identify state-specific keywords, such as important geographical names (e.g., Newport and Tillamook in OR vs. Puget [Sound], Olympia, Tacoma, Seattle, Olympic Peninsula, Grays [Harbor], and Everett in WA) and state agencies in charge of the transportation resiliency (e.g., ODOT, DOGAMI, and OEM in OR vs. DNR, WSF, and EMD in WA). The OR report places emphasis on *airports, airfield lighting, islanding*, and *backup power generation* in contrast to the WA report that highlights *railways, ferries*, and *companies*.

The insights discussed in this section provide a broader understanding of how regional differences and temporal factors shape the language and focus of disaster preparedness practical reports. The use of tf-idf can be applied to various domains, enabling researchers and policymakers to identify critical differences, shifts in focus, or areas requiring attention or alignment across different contexts.

### Sentiment analysis

#### Sentiment analysis—What is the portrayed sentiment of this corpus?

**Fig 6:**
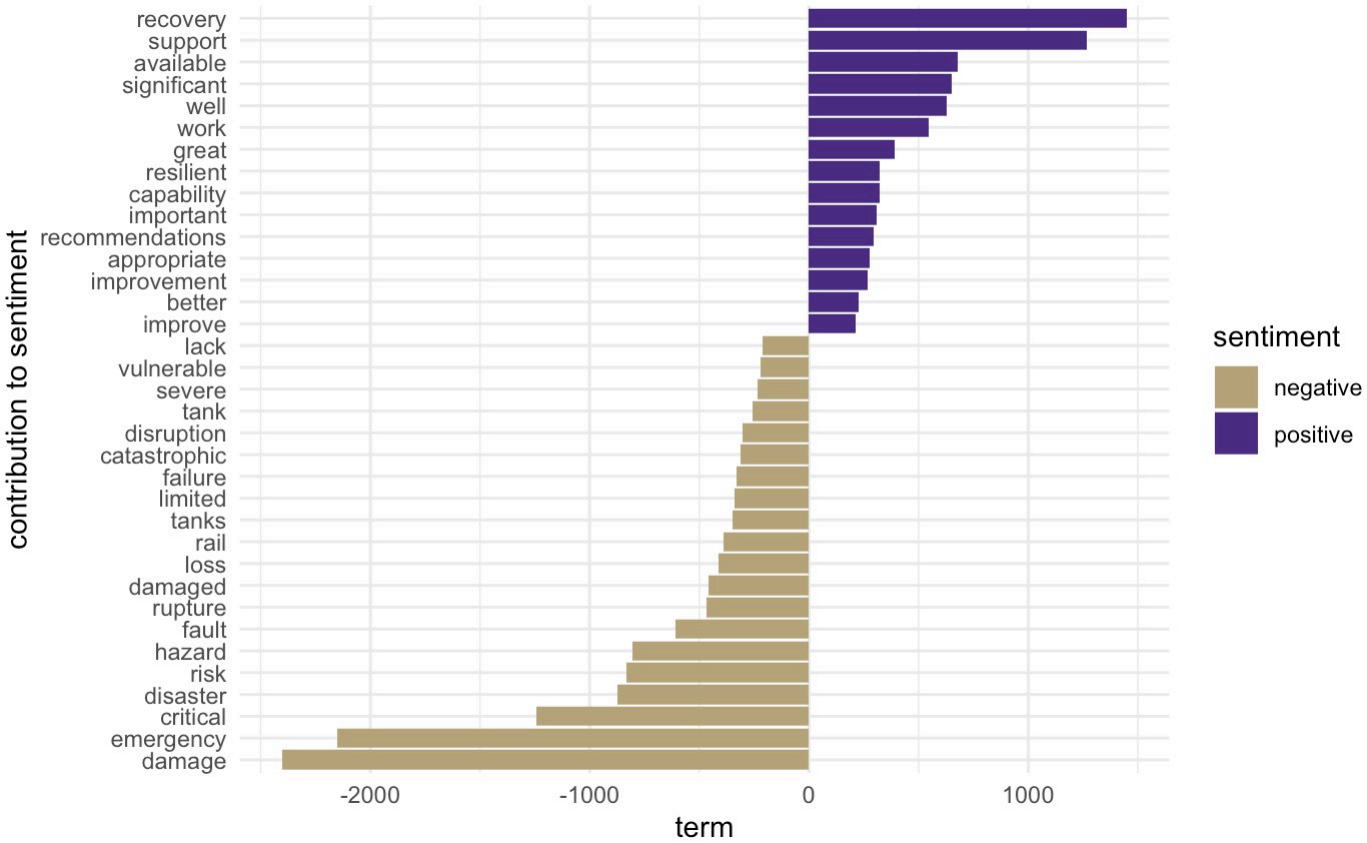
Sentiment analysis—Count of terms that have the greatest contribution to overall sentiment based on BING lexicon.

It is a known limitation of sentiment analysis that it can be challenging to distinguish between topic valence (e.g. the positive or negative sentiment associated with a particular topic) and the author’s position on the topic [54]. Fig 6 shows the predominantly negative sentiment of the corpus based on the Bing lexicon which could simply be representative of the adverse emotions and concerns associate with natural disasters. Conversely, it may prove to be a salient point with regard’s to the author’s choice of words, particularly given the prevalent fatalistic attitude towards the Cascadia Subduction Zone Earthquake of the public and, more often than not, emergency management practitioners (e.g., local emergency managers, and infrastructure managers). Further analysis is required to determine if this finding potentially calls for a paradigm shift in how we present a scenario catastrophic event in practice, to induce a more active and constructive engagement of society in the discussion of catastrophe preparedness. For example, future reports could focus more on positive themes (e.g., what are the benefits of mitigation and preparedness actions?) than negative themes. Future research could study the impact of overall negative sentiment or affect in emergency management practice reports, building upon the relevant risk communication literature that examined the relationship between negative affect and mitigation, preparedness, and protection behaviors [55–57].

#### Sentiment frequency—What emotions are portrayed most in a single document (Oregon Resilience Plan 2013?)

**Fig 7:**
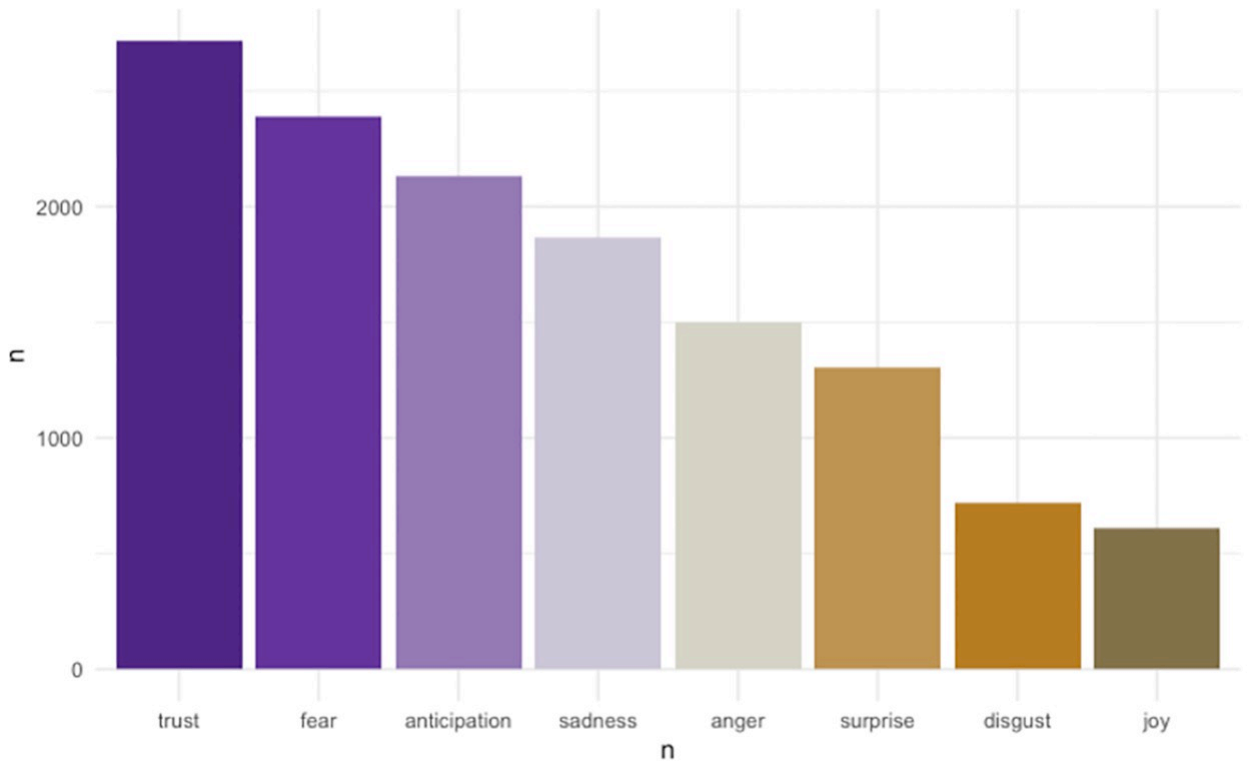
Sentiment frequency—Frequency of words by sentiment used in Oregon Resilience Plan 2013 according to NRC lexicon. Associates words with eight basic emotions in Plutchik’s wheel of emotions. [40].

Fig 7 offers a closer look into the NRC-based sentiment analysis that maps the document to eight basic emotions. Most frequent are ‘trust’-evoking words, followed by ‘fear’-evoking words. This analysis shows the potential value of sentiment analysis for better risk communication, where the impacts of trust and negative affect (e.g., fear, sadness, anger) are extensively studied [56].

The broader applicability of sentiment analysis in disaster preparedness reports lies in its ability to provide insight into the emotional tone and communication strategies used by authors. Identifying the predominant use of negative emotions, such as fear or sadness, could signal the need for a more balanced approach in risk communication. This tool can be applied across various fields where emotional engagement is key, helping to foster more constructive dialogues and encouraging proactive behavior by balancing negative sentiments with trust and optimism in future reports.

### Topic modeling and summarization

#### Topic modeling with 4 Topics Using Latent Dirichlet Allocation (LDA)—What are the most common topics discussed in the corpus?

When using LDA, we need to pre-specify the number of topics (i.e., clusters of terms) to be identified in the corpus. This number may be chosen based on domain knowledge or iteratively tested until reaching a reasonable number of topics (see Topic-Word Scores using Bidirectional Encoder Representations from Transformers (BERT)—what are the most common topics discussed in this corpus? for BERTopic’s ability to choose the number algorithmically). For this analysis, we tested several numbers of clusters (see S1 Appendix for details) and found a four-topic target to be the most useful for this corpus. Topic groups were interpreted by a co-author, knowlegeable in the field of disaster recovery, who examined commonalities and relationships among identified words to discern topics.

**Fig 8:**
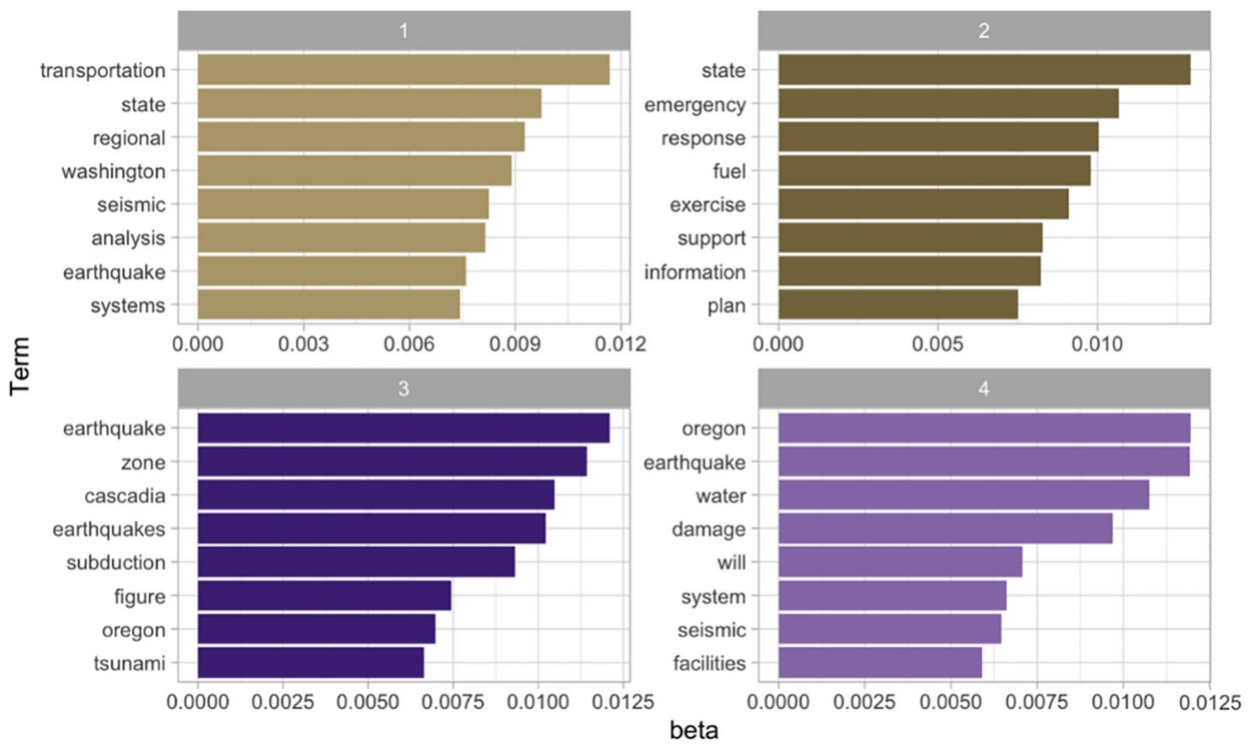
Topic modeling with 4 topics using LDA. User specified number of topics, documents categorized by shared terms and themes.

Fig 8 presents a subtle breakdown of the topics present in the corpus. Topic 1 singles out the importance of transportation systems in the regional and state (e.g., Washington)-level analysis of earthquake events. Topic 2 describes state-level emergency response plans that center around fuel, information, support, and exercise. Topic 3 identifies the core hazards (i.e., the Cascadia subduction zone earthquake and its induced tsunami), which tend to be illustrated through figures. Topic 4 centers around earthquake damage to water systems and facilities. This last topic’s common co-occurrence with ‘Oregon’ may indicate the perceived primary importance of the water system over other infrastructures to the resilience of Oregon.

#### Document-topic probabilities using Latent Dirichlet Allocation (LDA)—Which documents comprise the identified topics in Fig 8?

**Fig 9:**
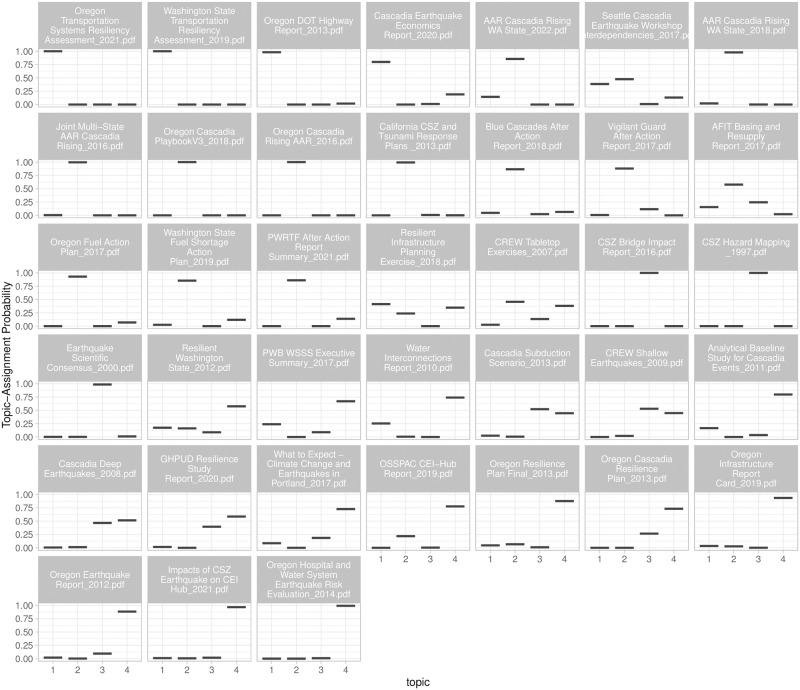
Document-topic probabilities using LDA. Quantitative representation of how each document in corpus relates to identified topic. (See Fig 4 in S1 Appendix for an alternative visualization of which documents contribute to which topics).

Fig 9 shows how the documents are distributed across the four topics identified in Fig 8. Some documents primarily represent a single topic group. For example, the first four documents are characteristic of Topic 1, which emphasizes regional and state transportation systems. This illustration also reveals that a document ‘Cascadia Earthquake Economics Report_2020.pdf’ that focuses on the Portland metropolitan region’s economics places much emphasis on transportation, helping readers to identify relevant documents. This type of visualization is generally helpful in summarizing the main themes of the corpus at a high level and in identifying similarly themed documents. For example, the documents in Topic 2 are generally state-level response exercise action-action reports or plans, whose cross-referencing is useful in practice, e.g., state-level benchmarking.

The potential for applying topic modeling reaches beyond disaster preparedness reports, benefiting any domain where analyzing large document sets for key themes is important. By clustering related documents and highlighting the most pertinent information, this tool streamlines knowledge management and allows professionals to grasp central themes efficiently.

#### Topic-word scores using Bidirectional Encoder Representations from Transformers (BERT)—What are the most common topics discussed in this corpus?

For this analysis, no preprocessing of documents is done, and stop words remain intact.

**Fig 10:**
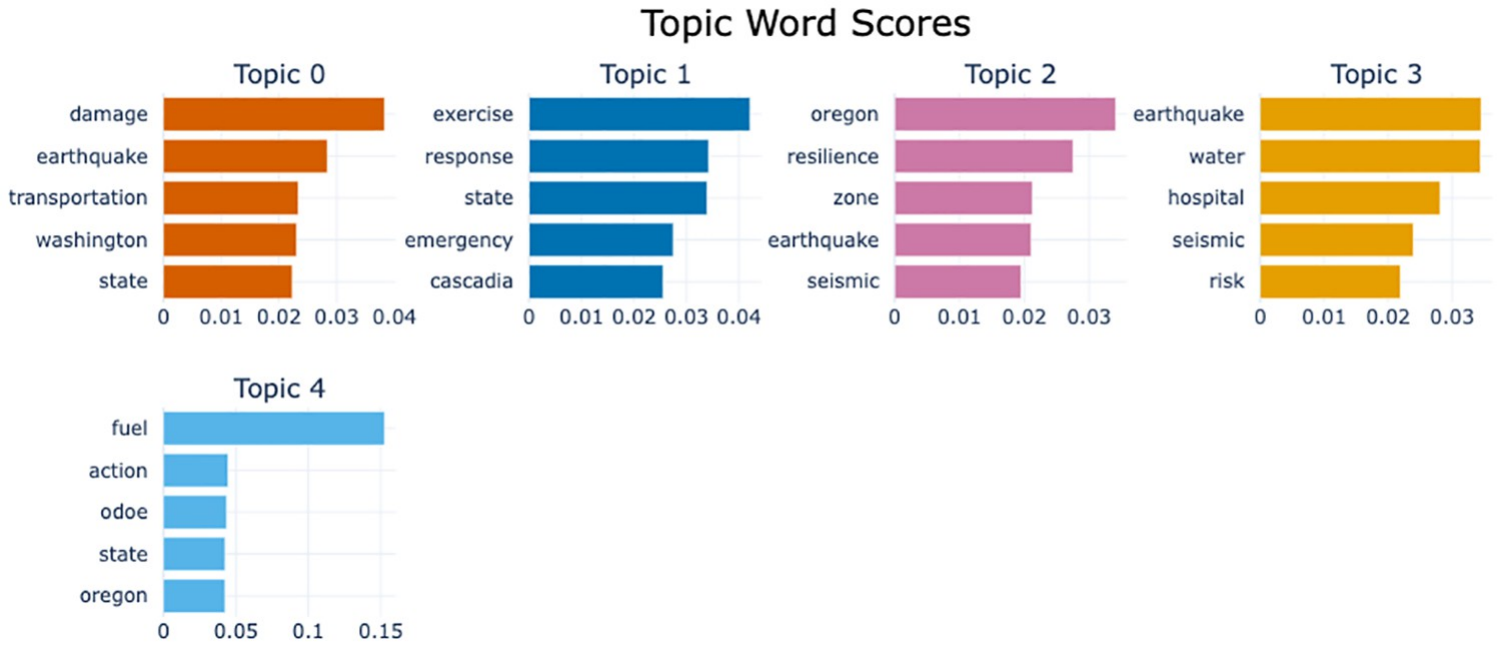
Topic-word scores using Bidirectional Encoder Representations from transformers. Term Frequency—Inverse Document Frequency (c-TF-IDF) Term Scores by Topic. The score reflects important terms within the identified topic cluster.

Fig 10 represents the class-based tf-idf of each representative topic identified by BERTopic, which leverages BERT and c-tf-idf reviewed in Bidirectional Encoder Representations from Transformers (BERT) and Generative Pre-trained Transformers (GPT). BERTopic allows for a flexible choice of the clustering algorithm to group related terms. We chose Hierarchical Density-Based Spatial Clustering of Applications with Noise (HDBSCAN) because of its ability to choose an optimal number of topics (i.e., clusters) by maximizing a measure of the overall stability of clusters [58]. The resulting five topics of BERTopic show good resemblance with the four topics chosen by the LDA in Topic Modeling with 4 Topics Using Latent Dirichlet Allocation (LDA)–what are the most common topics discussed in the corpus?:: Compare the BERTopic’s Topics 0, 1, 2, 3, and 4 with the LDA 4-Topic model’s Topics 1, 2, 3, 4, and 2, respectively. In other words, BERTopic found similar topics as LDA except for further breaking down LDA’s Topic 2 into Topics 1 and 4. In sum, LDA and BERTopic identified consistent topics within the corpus, demonstrating their robustness, although other corpora may lead to substantially different topics being identified by these two fundamentally different tools.

#### Short topic description using GPT-3.5—How can you summarize the content of representative documents in each identified topic in Fig 10?

GPT-3.5 is a version of OpenAI’s Generative Pre-trained Transformer model, which is specifically decisnged for natural language processing [59]. For each topic identified by the BERTopic model earlier, the short description in Table 2 and the one-paragraph summary in Table 3 provide a fuller picture of each topic’s representative documents in conjunction with each topic’s keywords in Fig 10. A summary of six outlier documents is provided as well. To create the short description in Table 2, we prompted the GPT model with “I have a topic that contains the following documents [DOCUMENTS]. The topic is described by the following keywords: [KEYWORDS]. Based on the above information, can you give a short label of the topic?” To create the paragraph summary in Table 3, we prompted the GPT model with the same [DOCUMENTS] and [KEYWORDS] prompt as with the short description but added a further prompt of “Based on the information above, please give a 100-word description of this topic”.

**Table 2:** Short topic description using GPT model. A brief topic description was generated using GPT-3.5 on the keywords and representative documents from the BERTopic model. Topic (-1) includes outliers. The count is the number of representative documents. Representation is the summarization of the representative documents.

Topic	Count	Representation
-1	6	Oregon’s Infrastructure and the Impact of Cascadia Subduction Zone Earthquake
0	8	Resilient Washington State’s Infrastructure Assessment and Recovery Framework
1	7	Cascadia Rising Exercise Response and Planning
2	7	Oregon Resilience Plan for Cascadia Subduction Zone Earthquake and Tsunami
3	6	Earthquake and Seismic Risk Assessment for Oregon’s Critical Infrastructure
4	4	Fuel Action Plan in Oregon for Emergency Water Supply

**Table 3:** Short topic description using GPT-3.5. A topic summary was generated using GPT-3.5 on the keywords and representative documents from the BERTopic model.

Topic	Count	Representation
-1	6	This topic focuses on the potential impact of a Cascadia Subduction Zone earthquake on various aspects of Oregon. The documents highlight the evaluation of infrastructures and the necessary upgrades to withstand such earthquakes. Additionally, there is information about Cascadia Subduction Zone earthquakes, including a scenario of a magnitude 9.0 earthquake. The topic also includes a summary of a Penrose Conference that discussed the tricentennial anniversary of the Great Cascadia Earthquake. Overall, the topic revolves around understanding the hazards posed by earthquakes in Oregon’s Cascadia Subduction Zone and the measures that can be taken to mitigate their effects on infrastructure and public safety.
0	8	This topic focuses on the assessment and analysis of the impacts of earthquakes, tsunamis, and other natural hazards on transportation infrastructure in the Pacific Northwest region, specifically in Washington State and Oregon. The documents mentioned highlight the importance of assessing the resiliency and vulnerability of regional bridge systems and port facilities in the face of potential seismic events, such as the Cascadia Subduction Zone earthquake. The analysis includes considerations of damage, emergency response scenarios, liquefaction risk, and the expected impacts on transportation systems and facilities. The goal is to develop strategies and plans to enhance the resiliency and preparedness of the infrastructure in the region.
1	7	This topic focuses on the planning and response efforts for a potential catastrophic earthquake and tsunami event in the Cascadia Subduction Zone. The documents mentioned include reports on the Cascadia Rising 2016 exercise, which involved multiple states and jurisdictions, including California and Washington. These reports discuss the coordination and management of information, operations, and resources at local, state, and federal levels. The goal is to enhance the capability and readiness of agencies and organizations involved in emergency response and recovery. The importance of public awareness, support, and collaboration in mitigating the impact of such a disaster is also highlighted.
2	7	This topic focuses on the Oregon Resilience Zone and its preparation for a potential earthquake in the Cascadia subduction zone. It discusses plans and strategies to enhance the resilience of buildings, infrastructure, and critical facilities, particularly in coastal areas. The topic also highlights the importance of mitigating fuel and oil-related risks, as well as the need for recovery and recovery models. Documents such as the Oregon Resilience Plan and the CEI Hub Mitigation Strategies provide insights into the state’s efforts to reduce risk and improve recovery in the event of a catastrophic earthquake and tsunami. The topic emphasizes the criticality of preparedness and response measures in mitigating the impact of such a natural disaster.
3	6	This topic focuses on the seismic risk and impact of earthquakes in the state of Oregon, particularly in relation to critical infrastructure such as hospitals and water systems. The documents highlight the economic analysis of a potential Cascadia subduction zone earthquake in the Portland metropolitan region, as well as the risks faced by Oregon’s critical energy infrastructure hub. It also includes a study on the earthquake risk evaluation of hospitals and water systems in the state. The topic covers various aspects such as the assessment of damage and risks, energy transmission systems, and the potential impact on the city of McMinnville and the Lincoln and Portland areas.
4	4	This topic focuses on fuel action and emergency response plans related to the state of Oregon. It includes keywords such as fuel, action, emergency, water, supply, agencies, and support. The mentioned documents highlight the importance of preparedness and response in the face of fuel shortages and the need for collaboration among water providers in the Portland metropolitan area. The topic also involves assessing information, planning, and coordinating efforts at the county, federal, and agency levels, with a primary mission to ensure an adequate fuel supply, especially during emergencies. The documents referenced were last revised in October 2017 and April 2019.

The generated summaries or representations are overall useful in gaining a quick overview of the topics. However, the artificial intelligence (AI)-generated wordings (i.e., AI’s best attempt to predict a sequence of words) in response to the prompt can be misleading. For example, Table 2’s Topic 0 representation can mislead uninformed readers to believe that all eight representative documents are part of the Resilient Washington State Initiative. Similarly confusing is Topic 2’s word choice “Oregon Resilience Plan,” which is specific to one document in 2013, to represent all seven documents in the topic. The longer-form representations in Table 3 are more informative overall but plagued by factual errors and misrepresentations (e.g., Topic 2’s “Oregon Resilience Zone” is the AI’s made-up term and Topic 4’s references to the documents’ last revised timestamps are not representative of all four documents). This issue of problematic AI products is an active research topic and at the center of ongoing societal debates [60]. Further clarification of these summaries may also be seen with the refinement of provided keywords, through the use of other keyword and sentence extraction algorithms such as TextRank [61] or Rapid Automatic Keyword Extraction [62].

### Synthesis of case study findings: Practical implications for emergency managers

In the section above, we discuss findings that are specific to each tool and their associated probing question. However, several overarching themes emerged from this analysis.

#### Communicating risk of M9 events

Our corpus of practical reports comprises publicly available reports aimed at raising community and professional awareness of the potential impacts of a Cascadia subduction zone M9 megathrust earthquake. Through our analysis, we have identified several opportunities for enhancing effective communication with the target audience. This includes uncovering latent sentiment expressed in the corpus (its predominant negativity shown in Fig 6) and observing in Term Frequency—Inverse Document Frequency of Whole Corpus—what are the unique/characteristic themes in each document of the corpus?. These findings underscore the importance for authors–whether practitioners or not–to be intentional in their preparation of practical reports, ensuring that they convey their message effectively. This requires considering the intended audience, the content being conveyed, and how it is presented. Effective communication of disaster preparedness information can have an impact on the way that communities, agencies, and citizens receive, process, and react to information [63].

#### Characterizing priorities

Many of the tools described in this work aided in understanding the priorities of the represented entities. Even during the initial phase of corpus development, our Google search revealed potential disparities among states. Oregon exhibited a more public-oriented approach to readiness, making its literature readily accessible and tailored towards individual preparedness. Conversely, Washington and California had fewer publicly available resources, and appeared to focus more on documenting the outcomes of readiness exercises. Furthermore, our analysis extracted the potential priorities of practitioners, as evidenced by prioritization of critical infrastructures depicted in Fig 2 and discerned differences in priorities between Washington and Oregon state-level resilience plans as shown in Fig 5). We also identified shifts in priorities over time, exemplified by the comparison of Cascadia Rising AARs in 2018 vs. 2022 in Fig 5). Understanding the priorities of stakeholders within the emergency management field is paramount, as it can hold critical implications for the efficacy and efficiency of essential response tasks (e.g. managing trade-offs and limiting conditions [64]). In harnessing the power of text mining, organizations can enhance communication and coordination efforts to ensure streamlined response efforts.

### Brief survey of emergency management researchers and practitioners

We asked over 100 emergency management researchers and practitioners for their voluntary, anonymous participation in reviewing our 9-page white paper (S1 File) and completing our 2-question online survey (Question 1—“What specific aspects of the tools did you find particularly useful? Why?” Question 2—“What challenges or limitations do you see in the tools shown in this document? Why?”) Of those surveyed, we received 5 responses from researchers and 22 responses from practitioners. Their feedback is summarized in Table 4. Though participants were not explicitly asked to describe their previous level of knowledge of the tools presented, many participants indicated that they had little to no prior experience with these tools.

**Table 4:** Summary of survey results from emergency management researchers and practitioners.

	**Researchers (*n* = 5)**
Specific tools found to be most beneficial	Term Frequency-Inverse Document Frequency of Select Pairs [Term Frequency—Inverse Document Frequency of Select Pairs—how do characteristic terms in one document compare to those of another related document?] Sentiment Analysis [Sentiment Analysis—what is the portrayed sentiment of this corpus?] Topic Modeling w/4 Topics Using LDA [Topic Modeling with 4 Topics Using Latent Dirichlet Allocation (LDA)—what are the most common topics discussed in the corpus?] Document Topic Probabilities Using LDA [Document-Topic Probabilities Using Latent Dirichlet Allocation (LDA)—which documents comprise the identified topics in Fig 8?]
Potential applications/benefits	Ability to understand global perspective for policy implementation Ability to understand prevalent framings and gaps in the documents that comprise the corpus Ability to objectively visualize documents to support equitable decision-making Ability to identify specific document(s) related to a given topic to facilitate efficient review
Limitations and challenges	Ease of use and training for implementation Accuracy of AI-generated summaries Need for expert input/review of results to ensure precision Corpus curation/development
	**Practitioners (*n* = 22)**
Specific tools found to be most beneficial	Term Relationships [Term Relationships—what are the predominant themes in this corpus?] Term Frequency-Inverse Document Frequency of Select Pairs Term Frequency—Inverse Document Frequency of Select Pairs—how do characteristic terms in one document compare to those of another related document?] Sentiment Analysis [Sentiment Analysis—what is the portrayed sentiment of this corpus?] Sentiment Frequency [Sentiment Frequency—what emotions are portrayed most in a single document (Oregon Resilience Plan 2013?)] Topic Modeling w/4 Topics Using LDA [Topic Modeling with 4 Topics Using Latent Dirichlet Allocation (LDA)—what are the most common topics discussed in the corpus?] Short Topic Description Using GPT-3.5 [Short Topic Description using GPT-3.5—how can you summarize the content of representative documents in each identified topic in Fig 10?]
Potential applications/benefits	Ability to account for and represent human emotion in corpus Ability to identify priorities, strengths, and weaknesses from AARs for action plans Ability to identify gaps in documents that comprise the corpus and gaps in coverage of relevant topics for the intended audience Ability to present comprehensible visualization Use of tool to determine where best to focus efforts (e.g. deep dive into certain document(s))
Limitations and challenges	Ease of use and training for implementation Accuracy of AI-generated summaries Corpus curation/development

Overall, researchers tended towards more traditional and proven text mining approaches such as LDA and tf-idf as opposed to the more state-of-the-art approaches, noting their hesitancy for trusting AI-based tools. They saw value in applying text-mining tools to visualize large amounts of data to support policy implementation and decision-making processes.

Practitioners found sentiment analysis and sentiment frequency to be the most intriguing and potentially useful tools for their work. More than half of the respondents noted that understanding the human emotion surrounding a document or corpus could contribute to their work and bring added value to their understanding of AARs, action plans, and assessments. Many respondents also noted that using feature analysis (tf-idf and term relationships) to compare various documents would be particularly helpful (as shown in Fig 5), as it allows a researcher to quickly see a shift or change in priorities over time.

Both researchers and practitioners expressed concerns about the cost-benefit tradeoffs in time and expertise needed to learn and implement new tools, and that results heavily rely on the quality of input, placing heavy emphasis on the development and curation of the studied corpus.

## Discussion

In this section, we discuss the strengths and limitations of our proposed approach, identify practical implications for researchers and practitioners, and discuss future work.

### Approach strengths and limitations

#### Practical reports as a corpus

This paper introduces a corpus of practical reports focusing on the Cascadia megathrust earthquake, analyzed using text mining tools. These reports offer authentic insights into planning and preparedness for this event, grounded in real-world experiences. By applying text mining tools to this corpus, researchers and practitioners can uncover valuable patterns, trends, and relationships, enhancing understanding and informing decision-making. Text mining enables a comprehensive analysis that goes beyond individual report examination, providing generalizable insights into the challenges and opportunities identified across the corpus.

One noted limitation is that the quality of text mining output depends on the quality of input data. We systematically collected practical reports for analysis but were restricted to publicly available documents. Furthermore, the structured nature of practical reports may limit the depth and richness of information compared to more unstructured sources. Despite these limitations, using tools that ensure equitable comparison, as demonstrated in Fig 5, and integrating domain-specific knowledge into the analysis can mitigate these challenges. Viewed through a different lens, pooling similar (though not identical) reports for analysis maintains confidentiality and proprietary information, while still extracting insightful thematic information from the corpus. Moreover, the structured format of these reports could be considered advantageous for future work, facilitating targeted mining for specific objectives, methods, results, and more.

#### Text mining as an approach

In this work, we introduce text mining as a powerful tool for efficiently extracting insights and knowledge from large volumes of textual data. It is flexible and scalable so that users can go beyond the surface level of the text and discover insights such as sentiment/emotion and latent topics, and can be applied to a variety of corpora. Overall, text mining can empower researchers and practitioners to harness the wealth of information contained within textual data, driving innovation, decision-making, and discovery across various fields and applications.

A recognized drawback of using text mining tools is the requirement for domain-specific and background understanding of the corpus to enhance interpretability and identify potential misrepresentations. Human language is inherently subjective and complex, making it essential for users to possess familiarity with the subject to navigate nuances effectively [65]. For example, one needs to be informed on the disaster preparedness field and its language to identify topic themes in topic modeling (e.g. recognizing an overarching theme encompassing the characteristic terms identified for each topic from a corpus) or decipher varying trends among compared reports (e.g. comparing WA vs. OR resilience plans. Domain knowledge is also required to validate results as shown in the usage of AI-assisted topic modeling (e.g. identification of “Oregon Resilience Zone” as a made-up term). Therefore, despite the promise of text mining and AI, in general, to help users learn from a corpus, those with experience in the field of the corpus are better positioned to benefit from the tools while guarding against their risks (e.g., misinformation from AI).

Furthermore, text mining, which offers the convenience of analyzing a large amount of text data computationally, is meant to *complement* more in-depth, targeted research tools, which tend to require more resources (e.g., subject-matter experts’ time to conduct qualitative analysis). For example, text mining-based exploratory analysis may help form specific research hypotheses and questions that may be probed through follow-up qualitative research.

#### Suite of tools

There are many text mining tools available, but in this work, we opted for ones that offered the most value for their usability and accessibility. The tools presented are easy to access through open-source programming languages (R and Python) utilizing user-friendly packages like tidytext, ggplot2, and BERTopic to conduct analysis and produce high-quality, concise visualizations. The use of these tools does require basic computer programming skills to implement, but with a modest investment of time, users can generate meaningful products tailored for use with any identified corpus.

Each text mining tool has its own set of strengths and limitations. Potential users may find Table 5 helpful in identifying the appropriate tools for their use.

**Table 5:** Strengths and limitations of text mining tools presented in this work. (+) denotes a strength, and (-) denotes a limitation.

	**Document and Corpus Feature Analysis**
Term Frequencies	(+) simple and straightforward approach of counting words’ frequencies to identify predominant themes in a corpus or document (−) lack of ability to determine reasoning/meaning behind included and/or missing terms (−) lack of calibration for the text of varying lengths (e.g. longer texts = greater frequency of words)
Term Relationships	(+) simple and straightforward tool to count the frequency of sequential and adjacent terms to identify patterns, themes, and relationships between terms (+) can be used for corpus or single document analysis (−) lack of ability to determine reasoning/meaning behind included and/or missing terms and relationships (−) lack of calibration for texts of varying lengths
Term Frequency-Inverse Document Frequency (TF-IDF)	(+) simple and straightforward tool to analytically identify the important words in each document by placing heavier weight on the words that are used less frequently in each document (+) allows for comparison of documents of varying length (+) easily meldable for use in other tools (−) can create unwieldy large vector spaces for documents with large vocabularies (−) does not account for similar words within vocabularies (−) lack of ability to determine semantic usage of terms
	**Sentiment Analysis**
Lexicon-based Sentiment Analysis	(+) easily accessible use of pre-established dictionaries to extract the emotional polarity of text (+) can be used for corpus or single document analysis (−) general-use lexicons may represent different sentiments than intended by the author—may require domain-specific lexicon
	**Topic Modeling and Summarization**
Latent Dirichlet Allocation (LDA)	(+) highly interpretable probabilistic model assigning documents to topics (−) number of topics must be specified—hyperparameters require tuning
BERTopic and GPT Models	(+) pretrained word embedding algorithm that allows for semantic relationships (+) results are easily interpreted (+) number of topics is determined automatically by an algorithm (−) word embeddings can make this model very large, requiring dimension reduction (−) carries general risks of AI (e.g., misrepresentation)

### Practical implications for researchers—Bridging the knowledge gap

The research-practice knowledge gap is not a novel challenge, but an enduring dilemma that transcends various disciplines, persisting despite ongoing efforts to bridge the divide. In this study, we present a novel approach—text mining of practical reports—to provide an objective representation of a practitioner’s perspective as embodied in these reports. While researchers are adept at understanding the research perspective based on their experience and expertise, they may lack some insight into the viewpoints of practitioners. By leveraging our research-based tools, even those with limited coding experience can utilize their seasoned expertise to identify discrepancies between research and practitioner perspectives.

### Practical implications for practitioners—A valuable addition to your toolkit

The integration of text mining presents practitioners with a valuable augmentation to traditional research tools and efficiently gain objective insights from diverse corpora. By employing this suite of tools, practitioners can access impartial insight into a corpus, which they may choose as a complement to conventional approaches such as literature review, environmental scans, landscape analysis, or use of a structured checklist. For example, emergency management consultants have the opportunity to customize the suite of tools provided, enabling them to seamlessly incorporate new corpora for analysis across different clients and alongside their conventional manual analyses. Unlike traditional approaches, which might be subjectively influenced by the reader’s presuppositions, text mining offers an objective lens. Busy practitioners, such as emergency managers, who may lack the time to review numerous reports individually, stand to benefit from the suite of text mining tools. While some initial preparatory work is necessary, once implemented, the suite facilitates a much quicker high-level overview of the corpus compared to traditional approaches (e.g., reading through the corpus). The incorporation of text mining tools empowers practitioners to elevate their research approaches and attain unbiased insights across various corpora. These insights can then be used as hypotheses to inform and spur further research, and then, once tested, encourage potential actions in practice.

### Future work

There are many opportunities to build upon this work. The presented toolkit can be expanded by exploring emerging tools in the fields of text mining, natural language processing, and AI in general. Further application of these tools to other corpora of practical reports can deepen our understanding of the advantages and limitations of the tools and expand their applicability by and accessibility to researchers and practitioners. Our proposed approach gives them the ability to extract overarching themes and novel insights from a corpus. In doing so, additional research questions are bound to emerge (e.g. the impact of sentiment on mitigation and preparedness behaviors). Insights gained from the presented tools can serve as a spark for other researchers and practitioners to formulate follow-on questions, objectives, and potential actions to advance their respective fields.

## Supporting information

S1 Appendix Appendix A. Additional insights gathered from the corpus.
(PDF)

S1 File Text mining of practical disaster reports. White paper used for survey.
(PDF)

S1 Fig (TIF)

S2 Fig (TIF)

S3 Fig (TIF)

S4 Fig (TIF)

S5 Fig (TIF)
