# Supplementary material for: Text mining of practical disaster reports: Case study on Cascadia earthquake preparedness
Source: PLoS One. 2025 Jan 7;20(1):e0313259. doi: 10.1371/journal.pone.0313259 (PMC11706397; doi:10.1371/journal.pone.0313259)
Supplement: S1 Appendix — Additional insights gathered from the corpus. (PDF) [file pone.0313259.s001.pdf]

Appendix A

In this manuscript we establish an approach for extracting meaningful insights from a corpus of practical reports. While our focus is on the approach, we were able to uncover many meaningful insights that may be useful to researchers and practitioners in the emergency management field. Below we highlight supporting information and additional insights that were gathered from our corpus.

Corpus Development

The Cascadia megathrust earthquake is expected to make an impact that will exceed any single state's emergency response capacity. As a result, the existing response plans assume extensive regional coordination as well as federal support. In this context, city- and county-level response plans are largely, if not fully, embedded in the state-level plans and thus excluded in our corpus to avoid unduly biasing it (e.g., due to a different number of cities and counties that have published their response plans, which overlap with their respective state-level plan). However, future studies that focus on local or municipal plans may carefully compile the relevant documents for further analysis (e.g., to identify any unique elements that stand out in local/municipal plans compared to the state/regional/federal plan) with a consideration that some local/municipal response plans for earthquakes and tsunamis may be absent or buried in a general (not a hazard-specific) emergency response plan.

Document and Corpus Feature Analysis

Term Frequencies

In [Term Frequencies - what are the predominant themes in this corpus?] we describe observations based on the frequency of a term found within the corpus. We note that water and transportation are the only two infrastructure systems that made it to the top 15 while 'electricity' and 'telecommunications' are noticeably absent. This trend's possible explanation is that the corpus frequently mentions 'fuel,' which is the primary energy source during the response phase. The corpus perhaps focuses more on the response than the long-term recovery, thereby placing 'fuel' above the electricity and

telecommunication infrastructures.

Additionally, we also note that ‘earthquake’ and ‘seismic’ are more frequently discussed than ‘tsunami.’ But, the frequency of ‘tsunami’ is not too far behind, indicating the practical reports’ attention on the coastal communities subject to tsunami. Similarly emphasized is ‘information,’ highlighting the practical interest around information (gathering and sharing).

Term Relationships

In [Term Relationships - what are the predominant themes in this corpus?] we illustrate a network of bi-gram terms used throughout the corpus to show the frequency of their use. In addition to the observations described in the manuscript, we also observe other notable pairs are water and transportation, frequently followed by ‘system(s),’ highlighting practical interests in the infrastructures as ‘system(s),’ which comprise multiple parts. In contrast, other infrastructure systems, such as ‘natural gas,’ ‘energy (sector),’ ‘public health,’ and ‘mass care,’ less frequently co-locate with ‘system(s)’ or ‘infrastructure.’

Other tools may be used for a more in-depth study. For example, the `widyr` package in `R` to calculate correlations among common pairs of words co-appearing within the same document section/chapter (not just in adjacency) and create a similar network graph for interpretation. This type of analysis can reveal whether practical reports tend to consider water and transportation systems together within the same section/chapter potentially because of their dependencies in vulnerability and resilience (e.g., water mains often co-locate with main roads, requiring coordinated repair efforts post-CSZ earthquake). Similarly, such in-depth studies can help identify whether practical reports are concerned about a specific infrastructure sector (e.g., energy) being operated primarily by the private sector, which may pose a challenge in coordinated recovery efforts post-CSZ (Chandra, Moen, & Sellers, 2016). Also, the word correlations may help reveal how practical reports consider (inter-)dependencies between infrastructure systems (e.g., Does the discussion of natural gas system co-appear with other dependent systems in the same section/chapter? Is the discussion of mass care placed in the context of public health infrastructure?). Similarly, the word correlations can help reveal more conceptual hubs, e.g., ‘Oregon’ (instead of ‘Washington’ or ‘California’), which connects liquid fuel, hospital, transportation systems, and water systems, perhaps due to the Oregon Resilience Plan (ORP)’s more

thorough consideration of the systems than other states' similar initiatives (Miles, 2018).

Term Frequency - Inverse Document Frequency

In [Term Frequency - Inverse Document Frequency of Whole Corpus - what are the unique/characteristic themes in each document of the corpus?], we use tf-idf to examine differences between three select pairs of documents. Below are the pairs used for analysis.

- Washington state CSZ event exercise AAR:

  `AAR Cascadia Rising WA State_2018.pdf`

  `AAR Cascadia Rising 2022 WA State_2022.pdf`

- State-level resilience planning:

  `Resilient Washington State_2012.pdf`

  `Oregon Resilience Plan Final_2013.pdf`

- State-level transportation systems study through the Regional Resiliency Assessment Program (RRAP):

  `WA State Transportation Resiliency Assessment_2019.pdf`

  `Oregon Transportation Systems Resiliency Assessment_2021.pdf`

In addition to the observations annotated in[Term Frequency - Inverse Document Frequency of Whole Corpus - what are the unique/characteristic themes in each document of the corpus?], we note additional findings when comparing Washington's CSZ Preparedness Exercises from 2016 to 2022. In CR16, the Department of Social and Health Services formed and led an ad-hoc mass care task force (TF) for *ESF* 6 (note that CR16's operations plan was organized around different TFs, such as JTF-WA and GTF). In CR22, "mass care services" became one of two essential core capabilities, along with "critical transportation." In contrast to CR16, CR22 placed much greater emphasis on mass care service and support for *shelters* and *food/nutrition* for *displaced* survivors and people with Access and Functional Needs (*AFN*).

Sentiment Analysis

In [Sentiment Analysis] we use the BING lexicon to examine the sentiment of the corpus and found a predominately negative sentiment throughout.

Figure 1 demonstrates the use of an alternate lexicon, Finn Årup Nielsen (AFINN) discussed in[Sentiment Analysis], for analysis of the corpus. Overall sentiment in this figure is represented by the frequency of the word used throughout the corpus multiplied by the assigned AFINN value. Although not as strong as those shown with the BING lexicon, the overall sentiment still tilts towards the negative end, being led by *catastrophic*, *damage*, and *loss*.

Figure 1: **Sentiment Analysis using AFINN Lexicon**

Figure 2 compares the Bing and NRC lexicons, discussed in [Sentiment Analysis] as well as the AFINN lexicon, for a single document–Oregon Resilience Plan 2013. The Finn Arup Nielsen (AFINN) lexicon assigns a score between -5 (negative) and 5 (positive) for each word in the corpus. Further analysis can be done using alternate lexicons that may be more specific to the given dataset(Nielsen, 2011). Oregon Resilience Plan is the longest document in the corpus making it most suitable to examine the differences between the lexicons. We removed stop words and then divided the document into 100 word sections (index) for easy comparison across the lexicons. The AFINN and Bing lexicons depict the document as overall negative. In contrast, the NRC lexicon sees the overall positive sentiment except for a few negative spots, which align with strongly negative spots identified in the previous two lexicons. A flipped, overall positive outlook of the NRC lexicon-based sentiment analysis is attributed to A) NRC's greater ratio of positive to negative words (2312/3324 = 69.6%) compared with AFINN (878/1598 = 54.9%) and Bing (2006/4783 = 41.9%) (Khoo & Johnkhan, 2018) and B) a high agreement of the sentiment assigned to each word between AFINN and Bing (98.8%) in contrast to NRC agreeing with AFINN at 83.1% and Bing at 80.9% (Ozdemir & Bergler, 2015).

Figure 2: **Comparison of Lexicons based on single document**

Figure 3: **Practical Report Topic Modeling with 2 Topics**

Figure 4: **Topic-Assignment Probabilities Using LDA.**

Topic Modeling

In [Document-Topic Probabilities Using Latent Dirichlet Allocation (LDA) - which documents comprise the identified topics in Figure 8?], we examine the use of Latent Dirichlet Allocation (LDA) to conduct topic modeling a 4-Topic model. We also conducted analysis of 2 topics. Figure 3 for the 2-Topic model suggests that the first dominant topic (on the left) focuses on hazards (e.g., earthquake, seismic, Cascadia [subduction] zone, tsunami) and damages with an emphasis on the State of Oregon and water (infrastructure) system. The second dominant topic (on the right) spans the state-level emergency response and recovery plans that center around fuel, information, support, and exercise. This topic grouping by the model naturally captures two "opposing forces": the first topic group (i.e., hazards and damages) represents what the nature presents to the society whereas the second topic group (i.e., response and recovery plans) represents what is planned by the society in anticipation of the first group. Additionally, there is a notable move of 'recovery' from this theme to another theme (i.e., the first group in the 4-Topic modeling) indicates that the concept of 'recovery' appears more frequently with the CSZ EQ, possibly carrying a more general sense than what the concept of 'response' does in the corpus.

Additionally, in [Document-Topic Probabilities Using Latent Dirichlet Allocation (LDA) - which documents comprise the identified topics in Figure 8?], we examine how documents are distributed across the 4 topics identified in Figure 8. In Figure 4 we take an alternate visual approach to see which group of documents make up each topic.

In [Topic-Word Scores using Bidirectional Encoder Representations from Transformers (BERT) - what are the most common topics discussed in this corpus?], we describe the use of HDBSCAN for topic modeling using BERTopic. In addition to topic representations we also exame the clustering hierarchy. A benefit of HDBSCAN is a more interpretable topic choice thanks to its ability to identify a clustering hierarchy, as shown in Figure 5. It reveals the closeness between Topics 1 and 4, as well as their relative distance from Topics 0, 2, and 3, all measured in terms of the cosine distance between topic

embeddings. Note that the closeness of Topics 0, 2, and 3 makes sense as they generally overlap with Topic 1 (hazards and damages) of the LDA's 2-Topic model, whose Topic 2 (state-level emergency response plan) generally covers BERTopic's Topics 1 and 4. In sum, LDA and BERTopic identified consistent topics within the corpus, demonstrating their robustness, although other corpora may lead to substantially different topics between the two fundamentally different techniques.

Figure 5: **Hierarchical Structure by Topic**

References

Chandra, A., Moen, S., & Sellers, C. (2016). *What role does the private sector have in supporting disaster recovery, and what challenges does it face in doing so?* Rand Corporation Santa Monica, CA.

Khoo, C. S., & Johnkhan, S. B. (2018). Lexicon-based sentiment analysis: Comparative evaluation of six sentiment lexicons. *Journal of Information Science*, *44*(4), 491–511.

Miles, S. (2018). Comparison of jurisdictional seismic resilience planning initiatives. *PLOS Currents Disasters*.

Nielsen, F. Å. (2011, March). Afinn. Richard Petersens Plads, Building 321, DK-2800 Kgs. Lyngby: Informatics and Mathematical Modelling, Technical University of Denmark. Retrieved from http://www2.compute.dtu.dk/pubdb/pubs/6010-full.html

Ozdemir, C., & Bergler, S. (2015). A comparative study of different sentiment lexica for sentiment analysis of tweets. In *Proceedings of the international conference recent advances in natural language processing* (pp. 488–496).
