# Supplementary material for: Text mining of practical disaster reports: Case study on Cascadia earthquake preparedness
Source: PLoS One. 2025 Jan 7;20(1):e0313259. doi: 10.1371/journal.pone.0313259 (PMC11706397; doi:10.1371/journal.pone.0313259)
Supplement: S1 File — White paper used for survey. (PDF) [file pone.0313259.s002.pdf]

Appendix B

Text Mining of Practical Disaster Reports

As you review this set of examples, please consider the following questions.

1. What specific aspects of the tools did you find particularly useful? Why?
2. What challenges or limitations do you see in the tools shown in this document? Why?

What motivated this work?

Throughout the last 20+ years, emergency managers across the Pacific Northwest have conducted planning conferences and emergency response exercises to increase awareness of potential impacts and build preparedness for an M9 Cascadia megathrust earthquake. Emergency management practitioners capture critical lessons learned, actions taken, and planning considerations from these practical field events in after-action reports, response plans, impact assessments, resiliency plans, and other practical reports. These documents are often lengthy and packed with dense information across a wide variety of topics and fields which can make them difficult for a reader to digest. Additionally, these documents are usually geared towards a specific disaster event, locality, or infrastructure which can discourage a reader from reviewing when it does not directly relate to their area of interest. **We propose examining these practical reports as an aggregated collection (corpus) using text mining** in order to increase accessibility and applicability to a wider audience.

What can text mining do?

In this work, we present a suite of text mining tools to efficiently examine this corpus of practical reports. The use of text mining tools can help uncover common trends and themes amongst the vast literature, distinct features that set apart one document from another, emerging patterns throughout a period of time, relationship and connections between documents or represented entities, or underlying emotion across the corpus. Examining this corpus, as an aggregate of information-rich individual reports, may reveal meaningful insights that may not have otherwise been uncovered by single documents alone.

3 categories of text mining tools that we explored in this work.

1. Document and Corpus Feature Analysis - examines word frequencies, relationships betweens word, and term frequency-inverse document frequency (tf-idf). These are simple and straightforward tools that aid the user in identifying predominant themes in the corpus.
2. Sentiment Analysis - tool used to identify the sentiment or emotion expressed in the document or corpus based on established lexicons (vocab or set of words specific to a domain or subject).
3. Topic Modeling - assigns a collection of texts into topics/themes using a traditional mathematical model and more recent AI tools like BERTopic and ChatGPT. Topic modeling allows the user to better understand the whole collection, aid in uncovering hidden themes from within the collection, assigning documents to discovered themes, or using these assignments to further organize or summarize the collection.

Document Collection Description

Our corpus consists of 38 publicly available reports representing emergency preparedness planning from Washington State, Oregon, and California as well as regional and federal level planning documents. A list of these reports can be found here. These reports include after-action reports, resiliency assessments, response plans, and planning scenarios related to the Cascadia Subduction Zone and M9 megathrust earthquake. The corpus encompasses both general reports as well as specific reports related to infrastructure recovery (e.g. water, power, transportation, and telecommunications). This corpus does not include reports related to hospital or healthcare emergency preparedness (unless coupled with another lifeline). Additionally, it is limited to technical, practical, and field reports and does not include academic research articles, news articles, blog posts, or non-technical articles (e.g. informational for the general public).

1. Document and Corpus Feature Analysis

Appendix B

Term Frequency (Figure 1) - Top 15 most frequently used single-word terms in corpus in decreasing order

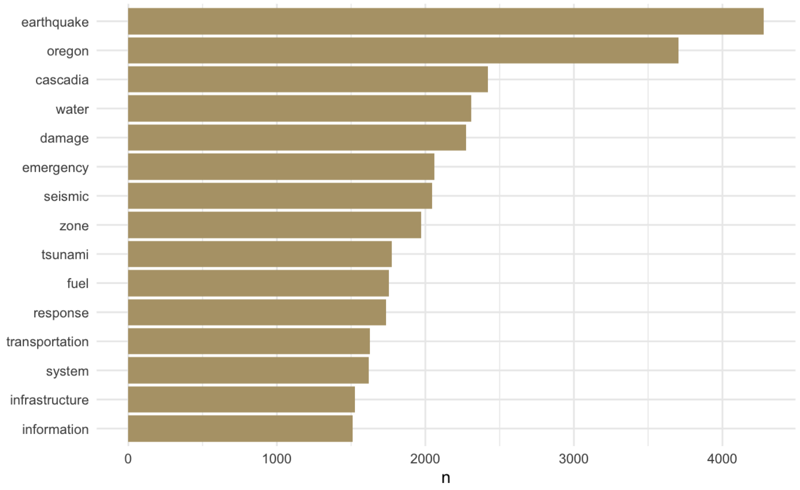

Probing Question: *what are the predominant themes in this corpus*?

Key Observations: Oregon is the second most frequently mentioned term while Washington did not make it to the list. Only two infrastructure systems (water and transportation) made it to the top 15. Both observations may warrant further investigation into the apparent 'gaps' to determine cause (e.g. if they represent an imbalance in preparedness efforts in practice).

Relationship Frequency (Figure 2) - Relationship between 2 sequential and adjacent terms occurring more than 200 times throughout the corpus. Opacity of the edge and background represent the frequency of the pair.

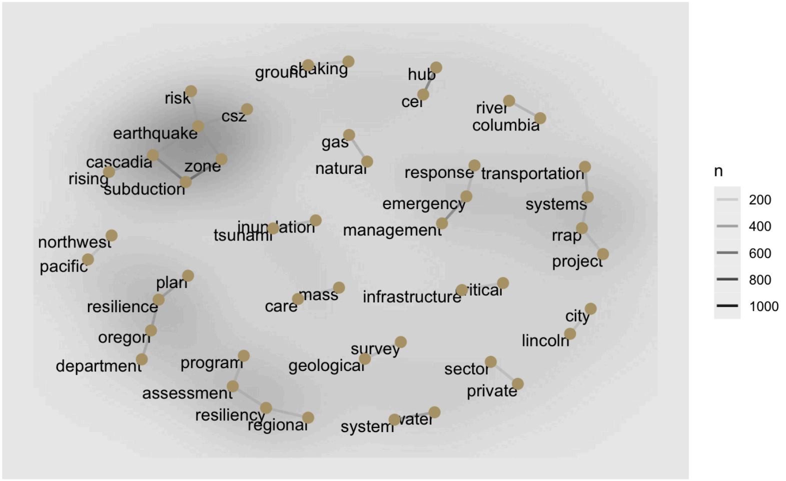

Probing Question: *what are the predominant themes in this corpus?*

Key observations: Figure 2 reveals more in-depth insights than Figure 1 into how practical reports use each term in its immediate context. (e.g. earthquake, Oregon, and Cascadia, commonly appear in specific contexts).

Term Frequency-Inverse Document Frequency (tf-idf) of Whole Corpus (Figure 3) - Top 11 characteristic terms that distinguish each document from the rest in the corpus

Appendix B

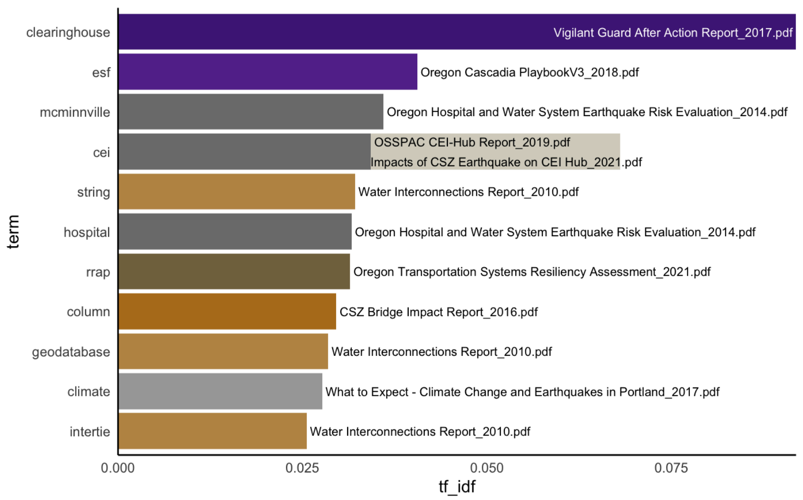

Probing Question: *what are the unique/characteristic themes in each document of the corpus?*

Key observations: only one or two documents pay attention to some terms widely considered critical in practice (e.g., clearinghouse, ESF [Emergency Support Function], geodatabase)

Term Frequency-Inverse Document Frequency of Select Pairs (Figure 4) - State level comparison of similar documents. Document pairs: 1) Washington state Cascadia Subduction Zone event exercise AAR in 2018 vs. 2022, 2) state-level resilience planning in Oregon vs. Washington, and 3) state-level transportation systems study in Oregon vs. Washington

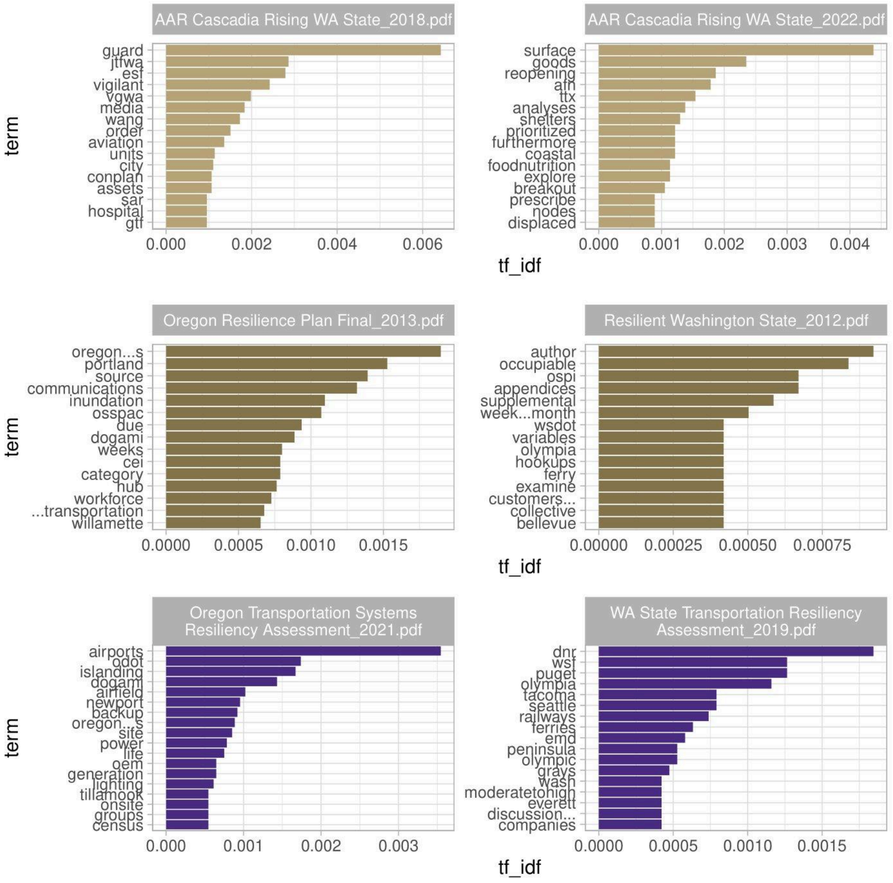

Probing Question: *how do characteristic terms in one document compare to those of another related document?*

Key observations: comparing the tf-idf of these couplets reveals insights into the shift in priorities over time (pair 1) or represented priorities from state to state (pairs 2 and 3).

2. Sentiment Analysis

Appendix B

Sentiment Analysis (Figure 5) - term count based on binary (negative/positive) categorization of terms

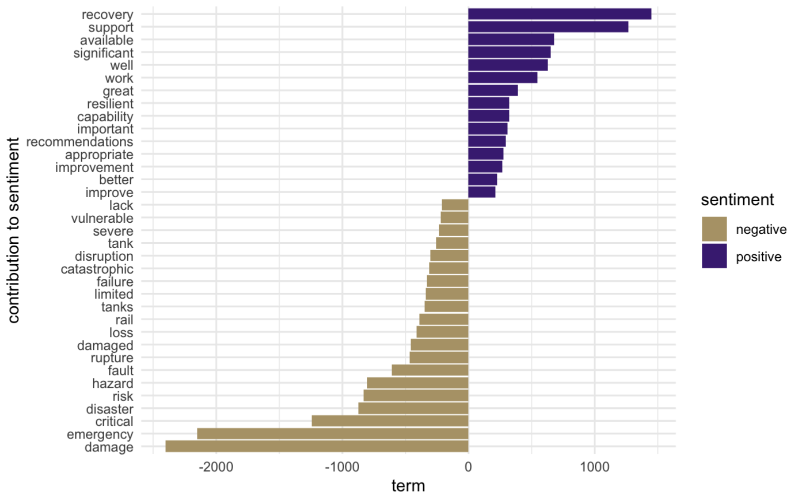

Probing Question: *what is the portrayed sentiment of this corpus?*

Key Observations: the predominantly negative sentiment of the corpus provides a fresh perspective about the cognitive burden on those who read practical reports in this corpus. This observation may lead authors to reconsider how we present a scenario catastrophic event in practice to induce a more active and constructive engagement of the public in discussion of catastrophe preparedness.

Sentiment Frequency (Figure 6) - mapping of single document to eight basic emotions

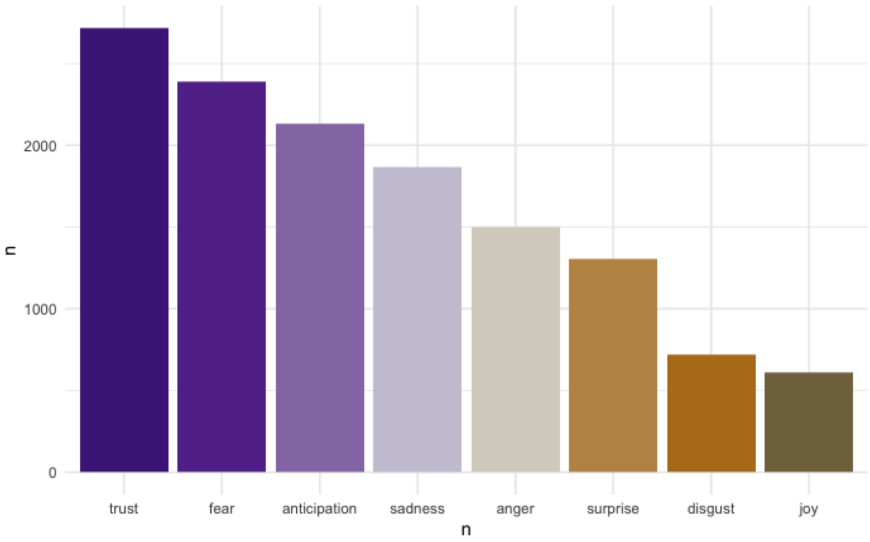

Probing Question: *what emotions are portrayed most in a single document (Oregon Resilience Plan 2013) ?*

Key Observations:
Most frequent are ‘trust’-evoking words, followed by ‘fear’ -evoking words. This analysis shows the potential value of sentiment analysis for better risk communications, where the impacts of trust and negative affect (e.g., fear, sadness, anger) are widely known.

3. Topic Modeling

Appendix B

Topic Modeling with 4 Topics Using Mathematical Modeling (Figure 7) - common topics in the corpus represented by frequently co-occurring terms

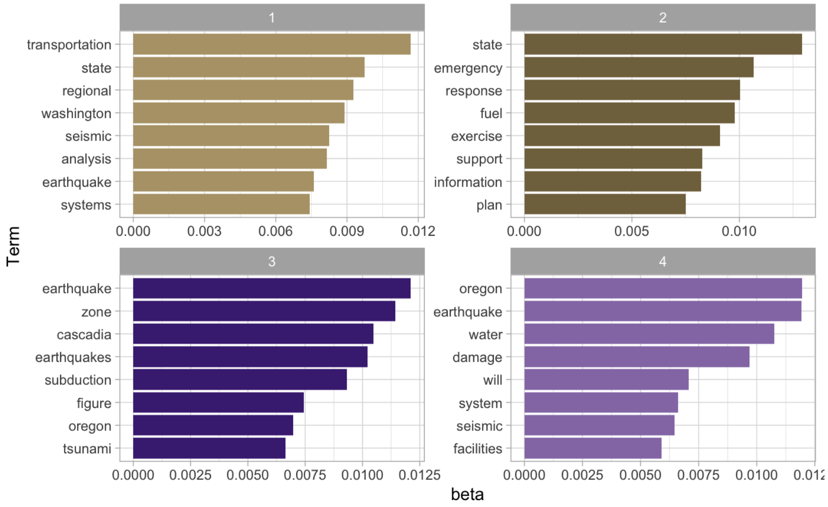

Probing Question: *what are the most common topics discussed in the corpus?*

Key Observations: Topic 1 emphasizes regional and state transportation systems. Topic 2 addresses the state-level emergency response plan around fuel, information, support, and exercise. Topic 3 identifies core hazards and Topic 4 centers around earthquake/seismic damage on water systems/facilities. The ability to specify topics with this method can aid with overall content understanding, information retrieval, and organization of documents.

Note: For this method, the number of topics is specified by the researcher based on domain knowledge and desired outcomes.

Document- Topic Probabilities (Figure 8) - topics (from Figure 7) distributed across the 38 documents in the corpus.

Appendix B

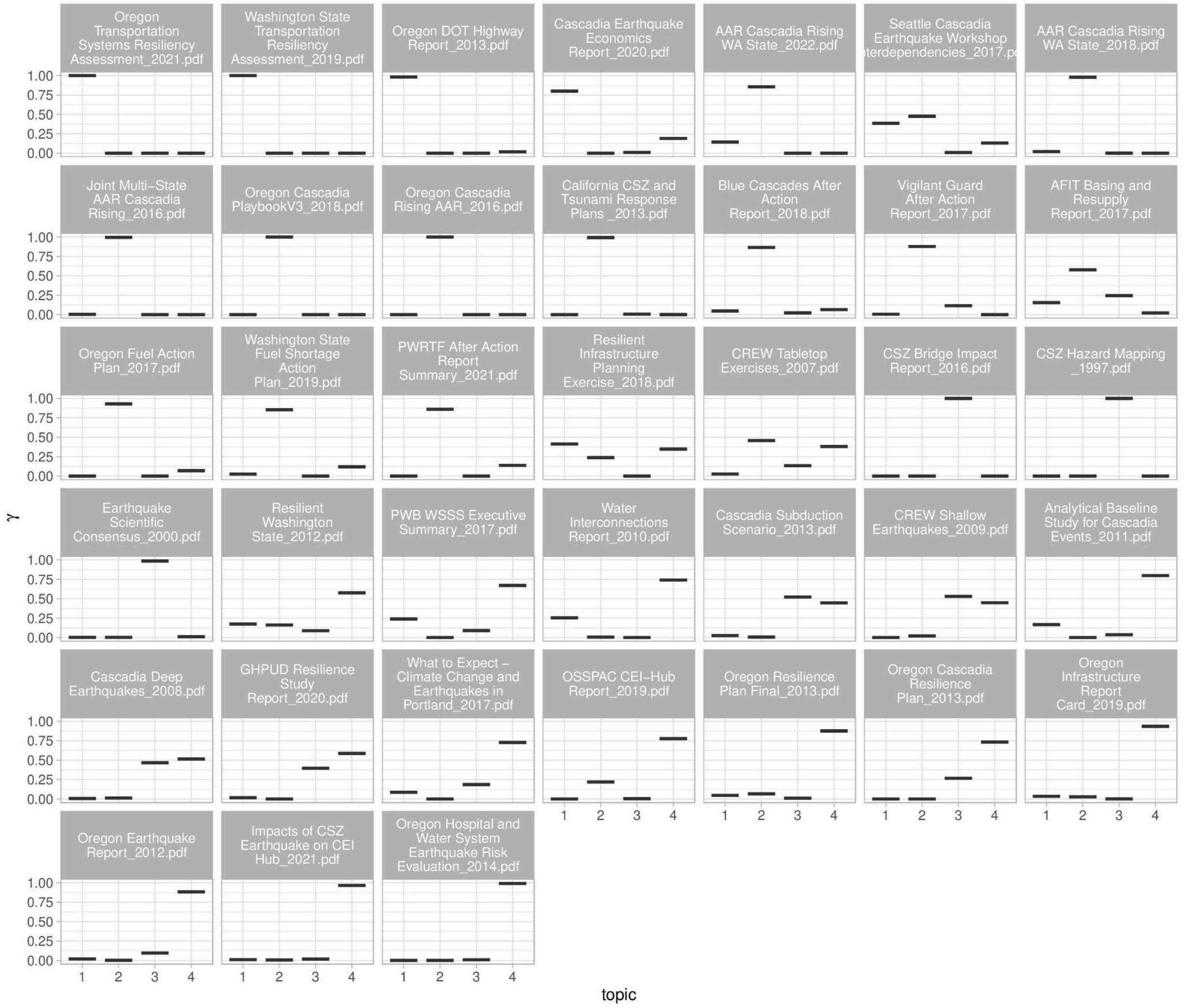

Probing Question: *which documents comprise the identified topics in Figure 7?*

Key Observations: Some documents primarily represent a single topic while others represent a more equal blend of several topics. This type of visualization is generally helpful in summarizing the main themes of the corpus at a high level and in identifying similarly themed documents. Additionally it helps readers to identify relevant documents if exploring a specific topic or theme.

Topic-Word Scores Using BERTopic (Figure 9) - important terms within the identified topic cluster using BERTopic (Bidirectional Encoder Representations from Transformers).

Appendix B

Probing Question: *what are the most common topics discussed in this corpus?*

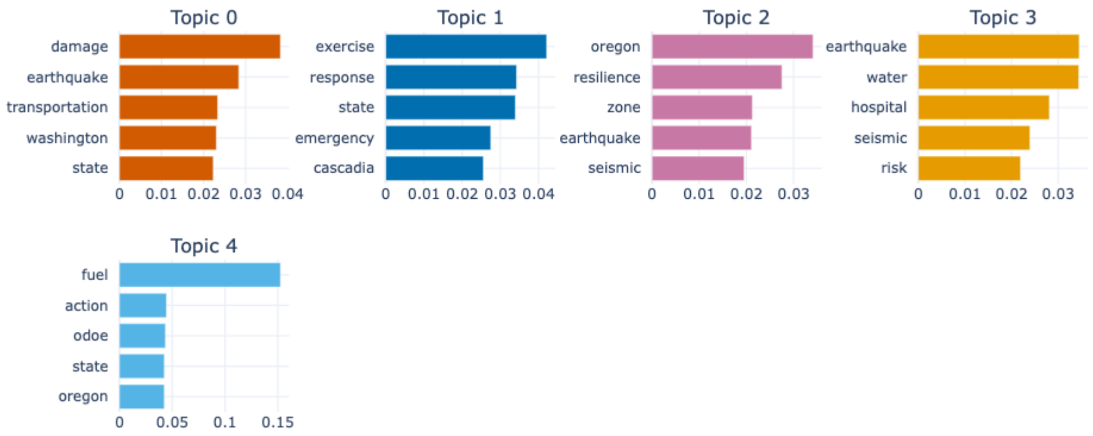

Key Observations: The resulting five topics (including Topic 0) of BERTopic greatly resemble those found using the mathematical model (Figure 7), but this more advanced AI tool is easier to use because it automatically chooses the number of topics (based on the closeness of terms between and within Topics).

Short Topic Description Using ChatGPT (Table 1) - Brief topic summary using ChatGPT generated from keywords and representative documents from BERTopic Model (Figure 9). Topic (-1) includes outlier documents. Count is the number of representative documents, and representation is the summarization of the representative documents.

Probing Question: *how can you summarize the content of representative documents in each identified topic?*

Topic	Count	Representation
-1	6	Oregon's Infrastructure and the Impact of Cascadia Subduction Zone Earthquake
0	8	Resilient Washington State's Infrastructure Assessment and Recovery Framework
1	7	Cascadia Rising Exercise Response and Planning
2	7	Oregon Resilience Plan for Cascadia Subduction Zone Earthquake and Tsunami
3	6	Earthquake and Seismic Risk Assessment for Oregon's Critical Infrastructure
4	4	Fuel Action Plan in Oregon for Emergency Water Supply

Topic Summaries Using ChatGPT (Table 2) - A longer topic summary (100 words) using ChatGPT

Appendix B

Topic	Count	Representation
-1	6	This topic focuses on the potential impact of a Cascadia Subduction Zone earthquake on various aspects of Oregon. The documents highlight the evaluation of infrastructures and the necessary upgrades to withstand such earthquakes. Additionally, there is information about Cascadia Subduction Zone earthquakes, including a scenario of a magnitude 9.0 earthquake. The topic also includes a summary of a Penrose Conference that discussed the tricentennial anniversary of the Great Cascadia Earthquake. Overall, the topic revolves around understanding the hazards posed by earthquakes in Oregon's Cascadia Subduction Zone and the measures that can be taken to mitigate their effects on infrastructure and public safety.
0	8	This topic focuses on the assessment and analysis of the impacts of earthquakes, tsunamis, and other natural hazards on transportation infrastructure in the Pacific Northwest region, specifically in Washington state and Oregon. The documents mentioned highlight the importance of assessing the resiliency and vulnerability of regional bridge systems and port facilities in the face of potential seismic events, such as the Cascadia Subduction Zone earthquake. The analysis includes considerations of damage, emergency response scenarios, liquefaction risk, and the expected impacts on transportation systems and facilities. The goal is to develop strategies and plans to enhance the resiliency and preparedness of the infrastructure in the region.
1	7	This topic focuses on the planning and response efforts for a potential catastrophic earthquake and tsunami event in the Cascadia Subduction Zone. The documents mentioned include reports on the Cascadia Rising 2016 exercise, which involved multiple states and jurisdictions, including California and Washington. These reports discuss the coordination and management of information, operations, and resources at local, state, and federal levels. The goal is to enhance the capability and readiness of agencies and organizations involved in emergency response and recovery. The importance of public awareness, support, and collaboration in mitigating the impact of such a disaster is also highlighted.
2	7	This topic focuses on the Oregon Resilience Zone and its preparation for a potential earthquake in the Cascadia subduction zone. It discusses plans and strategies to enhance the resilience of buildings, infrastructure, and critical facilities, particularly in coastal areas. The topic also highlights the importance of mitigating fuel and oil-related risks, as well as the need for recovery and recovery models. Documents such as the Oregon Resilience Plan and the CEI Hub Mitigation Strategies provide insights into the state's efforts to reduce risk and improve recovery in the vent of a catastrophic earthquake and tsunami. The topic emphasizes the criticality of preparedness and response measures in mitigating the impact of such a natural disaster.
3	6	This topic focuses on the seismic risk and impact of earthquakes in the state of Oregon, particularly in relation to critical infrastructure such as hospitals and water systems. The documents highlight the economic analysis of a potential Cascadia subduction zone earthquake in the Portland metropolitan region, as well as the risks faced by Oregon's critical energy infrastructure hub. It also includes a study on the earthquake risk evaluation of hospitals and water systems in the state. The topic covers various aspects such as the assessment of damage and risks, energy transmission systems, and the potential impact on the city of McMinnville and the Lincoln and Portland areas.
4	4	This topic focuses on fuel action and emergency response plans related to the state of Oregon. It includes keywords such as fuel, action, emergency, water, supply, agencies, and support. The mentioned documents highlight the importance of preparedness and response in the face of fuel shortages and the need for collaboration among water providers in the Portland metropolitan area. The topic also involves assessing information, planning, and coordinating efforts at the county, federal, and agency levels, with a primary mission to ensure an adequate fuel supply, especially during emergencies. The documents referenced were last revised in October 2017 and April 2019.

Key observations: The generated summaries/representations are overall useful in gaining a quick overview of the topics and distilling a large amount of information into a concise summary. However, the artificial intelligence (AI)-generated wordings (i.e., AI's best attempt to predict a sequence of words) can be misleading to the untrained eye as they were shown to have made up terms (e.g., "Oregon Resilience Zone" in Topic 2 in Table 2) and over-generalized some representative documents (e.g., for Topic 0 in Table 1, some representative documents are not part of the Resilient Washington State Initiative). This misrepresentation potential of AI is an active research topic and at the center of ongoing societal debates.

Share your feedback!

Appendix B

Above we described a suite of text mining tools applied to a corpus of practical reports. The tools presented here are not exhaustive, but demonstrate the opportunities for their use in a variety of different corpora. We invite you to provide your feedback at the link provided below on how you might see the use of these tools in your own work. Thank you in advance for your feedback!

https://forms.gle/ukL3sLLysquWthni6
