# Supplementary figures and images for: Text mining of practical disaster reports: Case study on Cascadia earthquake preparedness
Source: PLoS One. 2025 Jan 7;20(1):e0313259. doi: 10.1371/journal.pone.0313259 (PMC11706397; doi:10.1371/journal.pone.0313259)

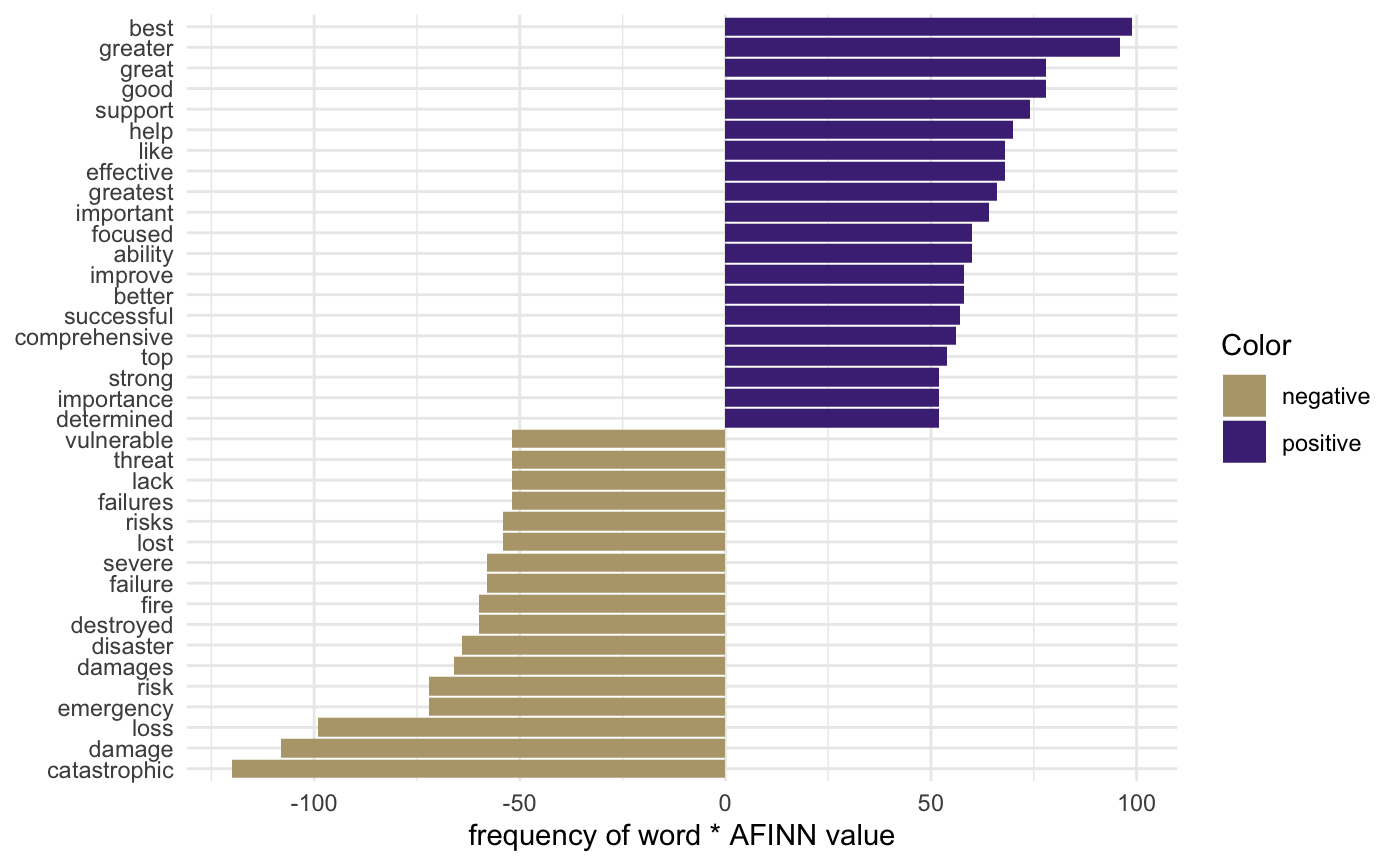

Supplement: S1 Fig — (TIF) [file pone.0313259.s003.tif]

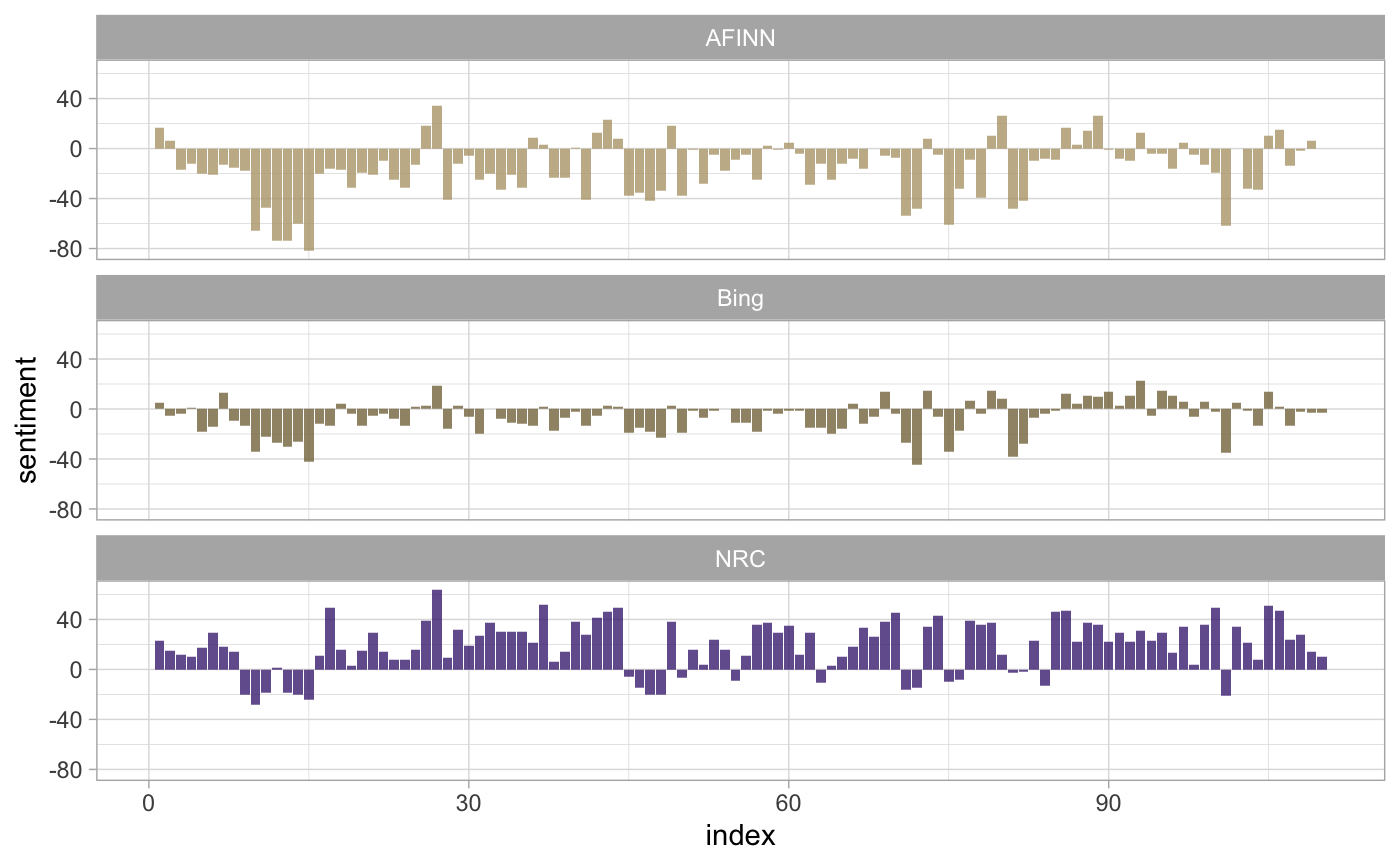

Supplement: S2 Fig — (TIF) [file pone.0313259.s004.tif]

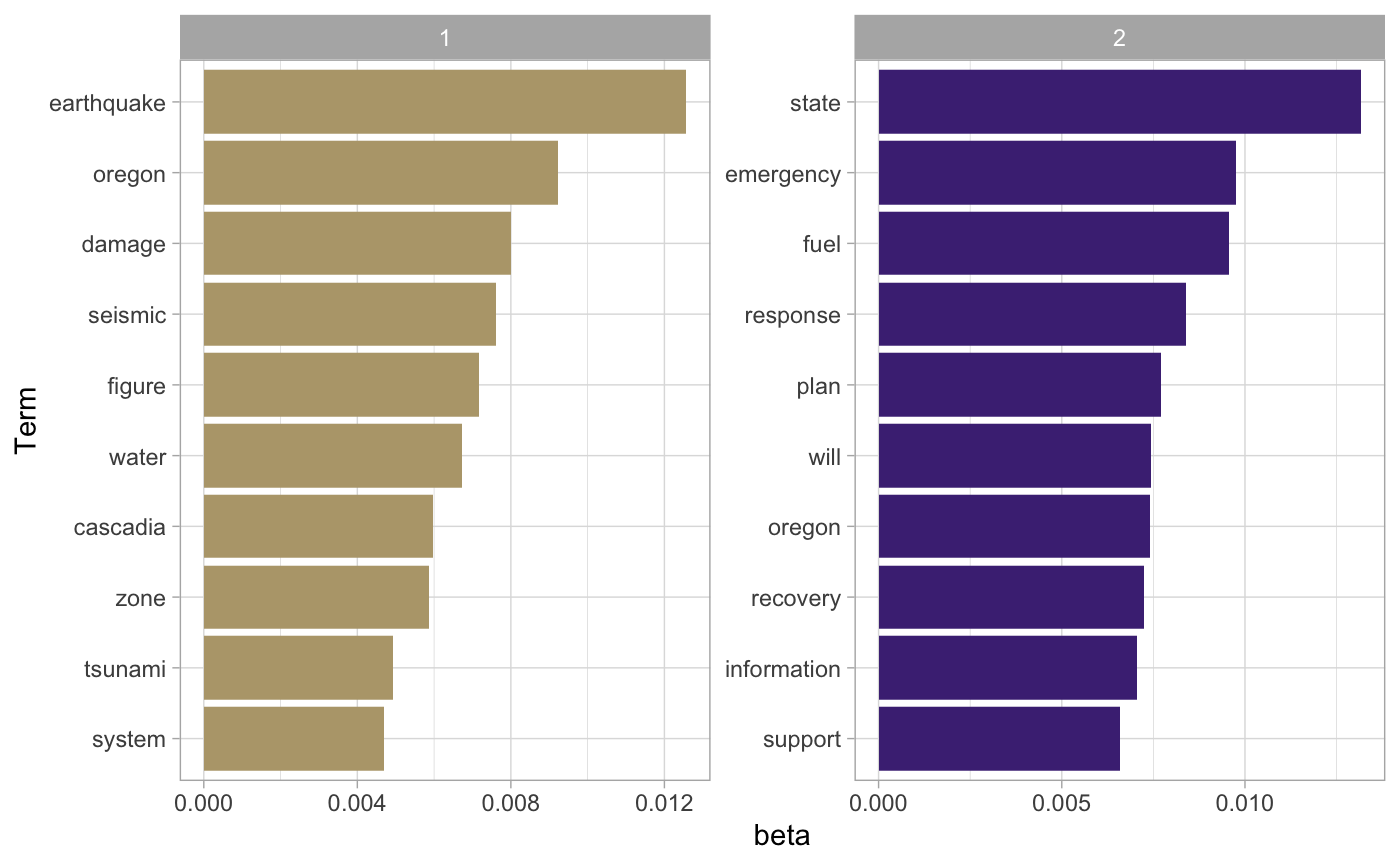

Supplement: S3 Fig — (TIF) [file pone.0313259.s005.tif]

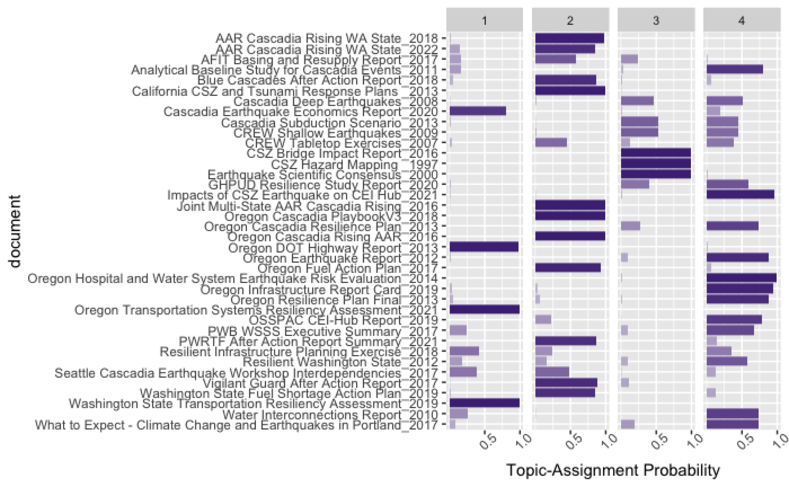

Supplement: S4 Fig — (TIF) [file pone.0313259.s006.tif]

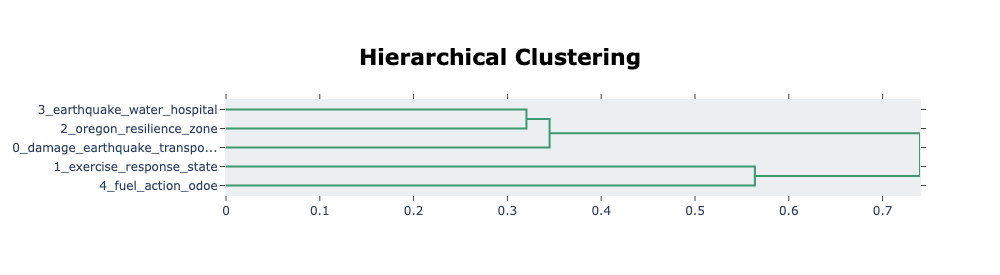

Supplement: S5 Fig — (TIF) [file pone.0313259.s007.tif]
